# Impaired autophagy bridges lysosomal storage disease and epithelial dysfunction in the kidney

**DOI:** 10.1038/s41467-017-02536-7

**Published:** 2018-01-11

**Authors:** Beatrice Paola Festa, Zhiyong Chen, Marine Berquez, Huguette Debaix, Natsuko Tokonami, Jenny Ann Prange, Glenn van de Hoek, Cremonesi Alessio, Andrea Raimondi, Nathalie Nevo, Rachel H. Giles, Olivier Devuyst, Alessandro Luciani

**Affiliations:** 1 Institute of Physiology and NCCR Kidney.CH, University of Zurich, 8057 Zurich, Switzerland; 2 Department of Nephrology and Hypertension, Hubrecht Institute and University Medical Center Utrecht, 3584 Utrecht, The Netherlands; 3 Division of Clinical Chemistry and Biochemistry, University Children’s Hospital Zurich, 8032 Zurich, Switzerland; 4 San Raffaele Scientific Institute, Experimental Imaging Center, 20132 Milan, Italy; 5 INSERM U1163, Université Paris Descartes, Institut Imagine, Hôpital Necker Enfants Malades, 75015 Paris, France

## Abstract

The endolysosomal system sustains the reabsorptive activity of specialized epithelial cells. Lysosomal storage diseases such as nephropathic cystinosis cause a major dysfunction of epithelial cells lining the kidney tubule, resulting in massive losses of vital solutes in the urine. The mechanisms linking lysosomal defects and epithelial dysfunction remain unknown, preventing the development of disease-modifying therapies. Here we demonstrate, by combining genetic and pharmacologic approaches, that lysosomal dysfunction in cystinosis results in defective autophagy-mediated clearance of damaged mitochondria. This promotes the generation of oxidative stress that stimulates Gα12/Src-mediated phosphorylation of tight junction ZO-1 and triggers a signaling cascade involving ZO-1-associated Y-box factor ZONAB, which leads to cell proliferation and transport defects. Correction of the primary lysosomal defect, neutralization of mitochondrial oxidative stress, and blockage of tight junction-associated ZONAB signaling rescue the epithelial function. We suggest a link between defective lysosome-autophagy degradation pathways and epithelial dysfunction, providing new therapeutic perspectives for lysosomal storage disorders.

## Abstract (web summary)

Nephropathic cystinosis is a lysosomal storage disease characterized by proximal tubular cell dysfunction. Here Festa and colleagues show that these lysosomal alterations lead to defective autophagic clearance of mitochondria and increased oxidative stress that, in turn, activates the transcription factor ZONAB leading to impaired cell differentiation.

## Introduction

The epithelial cells lining the proximal tubules (PT) of the kidney constitute a paradigm of effective communication between the environment and endomembrane compartments, allowing the reabsorption of essential nutrients. By processing incoming substances and recycling receptors and transporters at the apical plasma membrane, the endolysosomal system dictates cell differentiation, hence the maintenance of homeostasis^1,2^. The PT uptake accounts for ~ 80% of the clearance of small proteins and peptides, which are continuously filtered and completely reabsorbed by apical endocytosis involving the multi-ligand receptors, megalin, and cubilin^3^. Alterations in these transport processes lead to generalized PT dysfunction (an entity named renal Fanconi syndrome, RFS), causing urinary loss of solutes and low-molecular-weight (LMW) proteins, often complicated by dehydration, electrolyte imbalance, rickets, growth retardation, and development of chronic kidney disease (CKD). Such PT dysfunctions are typically encountered in congenital disorders due to defective endolysosomal transporters, particularly in nephropathic cystinosis^4^.

Cystinosis is a lysosomal storage disease (LSD) caused by recessive, inactivating mutations in the *CTNS* gene coding for the proton-driven transporter cystinosin that exports cystine out of lysosomes^5^. The loss of cystinosin causes an accumulation of cystine in tissues, leading to renal failure, diabetes, hypothyroidism, myopathy, and central nervous system deterioration. Infantile (MIM #219800) and juvenile (MIM #219900) forms of cystinosis represent a frequent cause of congenital PT dysfunction and RFS, most often complicated by CKD^6^. The only available strategy to counteract cystine storage is oral administration of cysteamine, which allows cystine to exit lysosomes. However, cysteamine treatment is hampered by side effects and poor tolerance, and it does not treat nor prevent PT dysfunction^6,7^. Thus, there is an urgent need to identify novel therapeutic strategies for this devastating disorder. Recent studies based on a *Ctns* mouse model that recapitulates multiple features of cystinosis^8^ have demonstrated that the loss of cystinosin is associated with aberrations of the endolysosomal compartment, and abnormal proliferation and dysfunction of PT cells^9^. Despite the identification of cellular defects associated with cystinosis in different models and cell systems^10^, a unifying mechanism linking loss of cystinosin, lysosomal dysfunction, and defective epithelial transport has not been deciphered.

In most mammalian cells, the endolysosomal system captures and degrades intracellular worn-out constituents through autophagy^11^. This homeostatic process is particularly active in PT cells, whose intense reabsorptive and transport properties require the maintenance of mitochondrial network^12^. The autophagy-mediated turnover of damaged mitochondria is required for protecting PT from acute tubular injury^13^, whereas deletion of essential autophagy genes damages PT cells through defective mitochondrial clearance and increased reactive oxygen species (ROS)^14^. Of note, accumulation of distorted mitochondria^15^ and of autophagy receptor SQSTM1/p62 has been described in kidney biopsies and urinary cells from cystinotic patients^16^, suggesting a possible involvement of autophagy. In addition, recent evidences show that cystinosin is a component of the lysosomal mammalian target of rapamycin complex1 (mTORC1)^17^, a hub that regulates autophagy-lysosome functions^18^ and nutrient transport in renal epithelial cells^19^. Altogether, these data suggest potential interactions between cystinosin function, the autophagy–lysosome degradation pathways, and the transport properties in PT epithelial cells.

In the present study, we decipher a pathway linking loss-of-function of cystinosin, lysosome–autophagy dysfunctions, mitochondrial oxidative stress, disruption of tight junction integrity, and activation of a signaling cascade causing epithelial cell dysfunction and loss of transport capacity. These insights offer new therapeutic strategies for treating epithelial dysfunction in nephropathic cystinosis and endolysosomal disorders.

## Results

### Loss of cystinosin alters lysosomal dynamics and autophagy

**Fig. 1:**
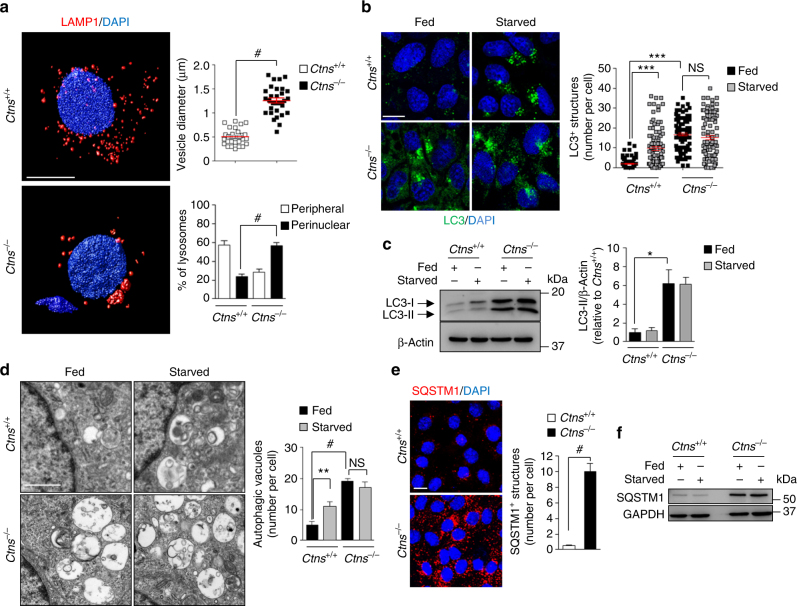
Abnormal lysosome dynamics and autophagy in CTNS-deficient PT cells. **a** Left: confocal microscopy and three-dimensional (3D) reconstruction of *Ctns* mPTCs labeled with anti-LAMP1 (red) antibody. Right: quantification of changes in vesicle size (top, each point representing the average size of LAMP1^+^ vesicles in a cell) and lysosome positioning (bottom, percent of perinuclear or peripheral distribution) (*n* = 30 cells pooled from 3 *Ctns* kidneys per group; two-tailed unpaired *t*-test, ^#^*P* < 0.0001 relative to *Ctns*^*+/+*^ mPTCs). **b**–**f** *Ctns* mPTCs were cultured in normal growth (Fed; 8 h) or nutrient-deprived cell medium (Starved; 8 h). **b** Representative confocal micrographs (left) and quantification (right) of numbers of LC3^+^ structures (green) in *Ctns* mPTCs (*n* = 100 cells pooled from three *Ctns* kidneys per group; each point representing the number of LC3^+^ vesicles in a cell; one-way analysis of variance (ANOVA) followed by Bonferroni’s posthoc test, ***P < 0.001 relative to *Ctns*^*+/+*^ mPTCs under fed conditions; NS, not significant.). **c** Western blotting and densitometric analyses of LC3 protein levels. β-Actin was used as a loading control. Two-tailed unpaired Student’s *t*-test, *P < 0.05 relative to *Ctns*^*+/+*^ mPTCs under fed conditions, *n* = 3 independent experiments. **d** Representative electron micrographs (left) and quantification (right) of numbers of autophagic vacuoles per cell (*n* = 10 micrographs per each condition; one-way ANOVA followed by Bonferroni’s posthoc test, **P < 0.01 and ^#^*P* < 0.0001 relative to *Ctns*^*+/+*^ mPTCs under fed conditions; NS, not significant). **e** Representative confocal micrographs and quantification of SQSTM1^+^ structures (red) in *Ctns* mPTCs (*n* = 100 cells pooled from three *Ctns* kidneys per group; two-tailed unpaired Student’s *t*-test, ^#^*P* < 0.0001 relative to *Ctns*^*+/+*^ mPTCs). **f** Representative western blotting of SQSTM1 in *Ctns* mPTCs. GAPDH was used as a loading control, *n* = 3 independent experiments. Plotted data represent mean ± SEM. Nuclei are counterstained with DAPI (blue). Scale bars are 10 μm in **a**, **b**, and **e**, and 2 μm in **d**. Unprocessed scans of original blots are shown in Supplementary Fig. 13

We first investigated the consequences of *Ctns* deletion on the lysosomal–autophagy pathways in epithelial cells. The loss of cystinosin, which was reflected by the accumulation of cystine in mouse kidneys and derived PT cells (mPTCs), induced a phenotype switch associating abnormal proliferation and apical dedifferentiation, leading to defective receptor-mediated endocytosis and urinary loss of LMW proteins in vivo (Supplementary Fig. 1a–g). These changes, which confirmed the validity of the *Ctns* mouse model and derived mPTCs^8,9^, were associated with a dramatic modification in lysosomal dynamics as evidenced by enlarged lysosomes, clustered into the perinuclear region (Fig. 1a and Supplementary Movies 1–2).

As the intracellular positioning of lysosomes coordinates autophagy in response to nutrient availability^20^, we tested whether the perinuclear clustering of lysosomes reflects changes in autophagy. We used mPTCs, because this primary culture system provides a particularly suited model to decipher the molecular mechanisms underpinning the endolysosome disorders within epithelial cells^21,22^. Autophagy was assessed by quantifying the conversion of the non-lipidated form of LC3-I to the lipidated, autophagosome-associated form LC3-II, and the numbers of LC3 vesicles^23^ in mPTCs cultured in nutrient-rich media (hereafter referred to as “fed”) or in nutrient-deprived conditions (hereafter referred to as “starved”). Compared with wild-type cells, *Ctns*^*−/−*^ cells showed higher numbers of punctate LC3 structures and steady-state levels of LC3-II, which did not further increase in starved conditions (Fig. 1b, c), as well as more electron microscopy (EM) structures compatible with autophagic vacuoles (Fig. 1d). Likewise, *Ctns*^*−/*^^−^ cells showed larger numbers of aggregates positive for the autophagy receptor SQSTM1 (Fig. 1e) and higher SQSTM1 protein levels, which did not further increase in starved conditions (Fig. 1f).

**Fig. 2:**
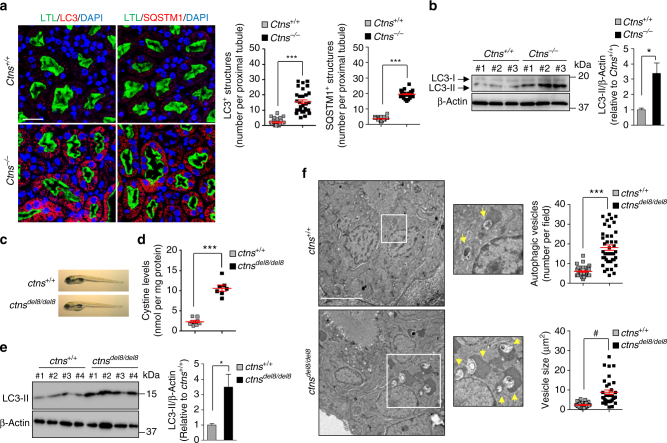
Cystinosin deficiency alters autophagy in proximal tubules of mouse and zebrafish kidneys. **a** Representative confocal micrographs and quantification of numbers of LC3^+^ (left panel; red) or SQSTM1^+^ structures (right panel; red) in LTL^+^ (green) proximal tubules in *Ctns* mouse kidneys (*n* = 30 proximal tubules pooled from three kidneys per group; each point representing the number of LC3^+^ or SQSTM1^+^ vesicles in a proximal tubule; two-tailed unpaired Student’s *t*-test, ***P < 0.001 relative to *Ctns*^*+/+*^kidneys). Nuclei counterstained with DAPI (blue). **b** Western blotting and densitometric analyses of LC3 protein levels in whole kidney lysates from *Ctns* mice (two-tailed unpaired Student’s *t*-test, *P = 0.029 relative to *Ctns*^*+/+*^ kidneys, *n* = 3 mice per group). **c** Representative images of *ctns*^*+/+*^ and *ctns*^*del8/del8*^ zebrafish embryos at 5 dpf and **d** quantification of cystine levels by HPLC in *ctns*^*+/+*^ and *ctns* ^*del8/del8*^ zebrafish embryos at 5 dpf (two-tailed unpaired Student’s *t*-test,***P < 0.001 relative to *ctns*^*+/+*^embryos, *n* = 8 *ctns*^*+/+*^ zebrafish and *n* = 7 *ctns* ^*del8/del8*^ zebrafish). **e** Western blotting and densitometric analyses of LC3 protein levels in pronephric tubules from 3-month-old *ctns*^*+/+*^ and *ctns* ^*del8/del8*^ zebrafish (*n* = 4 per group; two-tailed unpaired Student’s *t*-test, *P = 0.03 relative to *ctns*^*+/+*^ kidneys). **f** Representative electron micrographs and quantification of numbers and size of autophagic vacuoles in pronephric tubules of *ctns*^*+/+*^ and *ctns*^*del8/del8*^ zebrafish embryos at 5 dpf. White squares contain images at higher magnification. Yellow arrowhead indicates autophagic vacuoles. Number of autophagic vesicles: *n* = 43 (*ctns*^*+/+*^) and *n* = 42 (*ctns*^*del8/del8*^) randomly selected micrographs were pooled from 11 *ctns*^*+/+*^ and 12 *ctns*^*del8/del8*^ zebrafish. Average vesicle size: *n* = 32 (*ctns*^*+/+*^) and *n* = 33 (*ctns*^*del8/del8*^) randomly selected micrographs were pooled from 11 *ctns*^*+/+*^ and 12 *ctns*^*del8/del8*^ zebrafish. Two-tailed unpaired Student’s *t*-test, ***P < 0.001 and ^#^*P* < 0.0001 relative to *ctns*^*+/+*^ zebrafish. β-Actin was used as a loading control in **b** and **e**. Plotted data represent mean ± SEM. Scale bars are 50 μm in **a** and 5 μm in **f**

The accumulation of LC3-marking autophagosomes and SQSTM1-positive aggregates was consistently detected in the PT of *Ctns*^*−/*^^−^ kidneys (Fig. 2a, b). To further explore the consequences of cystinosin deletion in vivo, we established a novel *ctns* knockout zebrafish model using the TALENs (transcription activator-like effector nucleases) technique (Supplementary Fig. 2a–c). One mutant zebrafish line showed an 8 bp TALEN-driven deletion (*ctns*^*del8/del8*^), resulting in a premature stop codon within exon 3 of *ctns*, which would result in a truncated protein deprived of the transmembrane domains of cystinosin. Microinjection of wild-type (but not mutant) human *CTNS* messenger RNA decreased the cystine storage in *ctns-*deficient embryos to the same extent than cysteamine treatment (Supplementary Fig. 2d). The protein levels of LC3-II and the numbers of autophagic vesicles (AVs) were remarkably increased in cystine-accumulating pronephric tubules in *ctns*-deficient zebrafish (Fig. 2c–f), demonstrating the evolutionary conservation of this connection. These results demonstrate that the deletion of cystinosin alters lysosomal dynamics and autophagy in PT cells, both in vitro and in vivo*.*

### Cystinosin deficiency perturbs autophagic clearance in PT cells

**Fig. 3:**
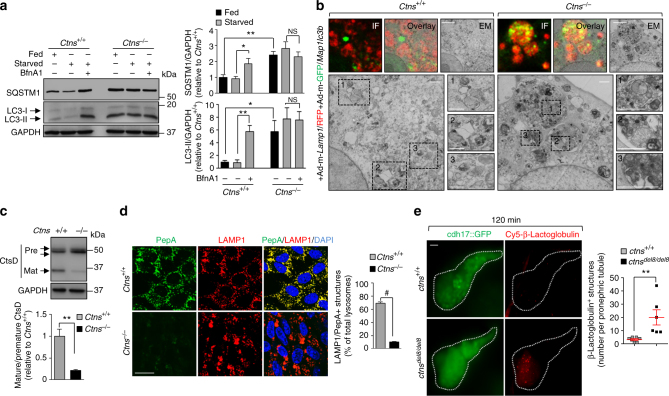
Cystinosin deficiency delays the clearance of autophagosomes by impairing lysosome function. **a** Western blotting and densitometric analyses for LC3 and SQSTM1 protein levels in *Ctns* mPTCs cultured in fed or starved medium in the presence or in the absence of 250 nM BfnA1 for 4 h (*n* = 3 independent experiments). Two-tailed unpaired Student’s *t*-test, **P < 0.01 (SQSTM1) and *P < 0.05 (LC3) relative to fed *Ctns*^*+/+*^ mPTCs, and *P = 0.05 (SQSTM1) and **P = 0.009 (LC3) relative to starved *Ctns*^*+/+*^ mPTCs. **b** Correlative light-electron microscopy (CLEM) in *Ctns* mPTCs co-transduced with RFP-tagged *Lamp1* (*LAMP1*/RFP, red) GFP-tagged *Map1lc3b* (*Map1lc3b*/GFP, green) bearing adenoviral particles for 2 days. The colocalization of GFP-LC3 and RFP-LAMP1 was monitored by confocal microscopy. Selected cells were further processed and serial sections analysed by electron microscopy. Dotted black squares contain images at higher magnification. Scale bars are 2 μm (top panel) and 500 nm (bottom panel). **c** Western blotting and densitometric analyses of CtsD protein levels in *Ctns* mPTCs; two-tailed unpaired Student’s *t*-test, **P < 0.01 relative to *Ctns*^*+/+*^ mPTCs, *n* = 3 independent experiments. **d** *Ctns* mPTCs were loaded with Bodipy-FL-PepA (1 μM, for 1 h at 37 °C, green), immunostained with anti- LAMP1 antibody (red) and analysed by confocal microscopy. Quantification of numbers of PepA/LAMP1^+^ structures (in percentage of total lysosomes; *n* = 5 randomly selected fields per condition, with each containing ~ 20–25 cells; two-tailed unpaired Student’s *t*-test, ^#^*P* < 0.0001 relative to *Ctns*^*+/+*^ mPTCs). **e** *ctns* zebrafish embryos expressing *cdh17*::GFP (green, pronephric tubule marker) were injected at 5 dpf with Cy5-tagged-β-lactoglobulin. At 120 min after the injection of the tracer, zebrafish embryos were imaged using light sheet fluorescent microscopy. A similar rate of internalization of Cy5-tagged-β-lactoglobulin was observed at 20 min in both *ctns*^*+/+*^ and *ctns*^*del8/del8*^ zebrafish embryos, validating the uptake of the tracer by pronephric tubules. Representative micrographs (left) and quantification (right) of numbers of β-lactoglobulin^+^ structures (red) in *ctns* zebrafish pronephric tubule (*n* = 6 zebrafish per group; two-tailed unpaired Student’s *t*-test, **P = 0.01 relative to *ctns*^+/+^ zebrafish). GAPDH was used as loading control in **a** and **c**. Plotted data represent mean ± SEM. Nuclei are counterstained with DAPI (blue). Yellow indicates colocalization. Scale bars are 10 μm in **d** and 50 μm in **e**

An increased number of AVs may arise from stimulation of autophagosome biogenesis or from alteration of their degradation by lysosomes. To discriminate between these two possibilities, we treated *Ctns* mPTCs with Bafilomycin A1 (BfnA1), a proton pump inhibitor that prevents lysosome degradation and thus increases autophagic cargoes and substrates exclusively when autophagy is active^23^. Treatment with BfnA1 heightened the amounts of LC3-II and SQSTM1 in nutrient-deprived *Ctns*^*+/+*^ cells, whereas it did not change the already elevated levels of these proteins in nutrient-deprived *Ctns*^−/−^ cells (Fig. 3a).

We next performed a pulse-chase assay to monitor the degradation of resting autophagosomes in *Ctns* mPTCs. Cells were starved to form autophagosomes and then treated with the class III phosphoinositide 3-kinase (PI3K) vacuolar protein sorting 34 (Vps34) kinase inhibitor SAR-405^24^ to prevent the formation of new autophagosomes. We validated the selectivity of SAR-405 by determining the intracellular phosphatidylinositol-3-phosphate pools (PtdIns-3P, the end-product generated by Vps34 kinase activity) in mPTCs (Supplementary Fig. 3a). The degradation of the formed autophagosomes following SAR-405 treatment was tracked by time-lapse confocal microscopy: in contrast to the autophagic-mediated degradation observed in starved *Ctns*^+/+^ cells, the *Ctns*^−/−^ cells retained almost all the formed autophagosomes (Supplementary Fig. 3b, c). Upstream events regulating autophagosome biogenesis, such as conjugation (ATG7) and phagosome formation (ATG16L + vesicles), including Beclin1, remained unchanged in control and *Ctns*^−/−^ PT cells (Supplementary Fig. 4a–c). Taken together, these results indicate that the accumulation of autophagosomes in *Ctns*^−/−^ cells results predominantly from a slower autophagosome clearance rather than augmented biogenesis.

### Defective lysosomal function impedes autophagy in *Ctns*^−/−^ cells

A blockade in LC3-II degradation can occur at any step after autophagosome formation and can be induced by delayed trafficking of autophagosomes to lysosomes and/or reduced fusion between both compartments^25^. We thus explored whether the delayed autophagosome clearance in *Ctns*^−/−^ cells might be caused by an impaired autophagosome delivery to lysosomes. To test this hypothesis, we examined the subcellular distribution of LC3-marking autophagic structures and LAMP1-labeled lysosomes in response to short incubations with non-saturating concentrations of BfnA1 (50 nM for 1 h)^26^. Confocal microscopy analysis showed that cystinosin deletion did not decrease the colocalization of LC3 and LAMP1-labeled lysosomes in BfnA1-treated PT cells but rather increased the number of large LAMP1 + vesicles filled by LC3 in *Ctns*^−/−^ PT cells (Supplementary Fig. 5a). Electron tomography microscopy and three-dimensional (3D) reconstructed tomograms confirmed the existence of enlarged, single-membrane structures engulfed with partially digested cellular debris in *Ctns*^−/−^ cells (Supplementary Fig. 5b and Supplementary Movie 3). Accumulation of autophagic material in autolysosomes of *Ctns*^−/−^ cells was also confirmed by correlative light-EM, where GFP-LC3-positive autophagosome containing cellular constituents coalesced within enlarged, single-membrane RFP-LAMP1-positive organelles (Fig. 3b), indicating that the transport and/or autophagosome-lysosome fusion are not compromised.

One mechanism by which the autophagic cargo clearance might be impeded is defective lysosomal degradation capacity^11^. To substantiate the delayed autophagosome degradation in *Ctns*^−/−^ cells, we analyzed trafficking, processing, and maturation of lysosomal cathepsins. Western blot analyses of cathepsin-D (CtsD) showed a decreased proteolytic generation of the 32 kDa mature CtsD in *Ctns*^−/−^ compared with *Ctns*^+/+^cells (Fig. 3c). We next tested the lysosomal CtsD activity by incubating mPTCs with Bodipy-FL-PepstatinA (PepA), a fluorescence-tagged PepA that binds to the active site of CtsD in acidic lysosomes^22^. Although the majority of lysosomes were co-stained with PepA-labeled CtsD in *Ctns*^+/+^cells, the number of PepA-labeled vesicles and the colocalization of active CtsD with LAMP1 were substantially lower in *Ctns*^−/−^ cells (Fig. 3d). These changes in lysosome function were observed despite normal trafficking of CtsD between Golgi and LAMP1 compartments (Supplementary Fig. 5c, d). Similarly, the lysosome-based processing of the LMW β-lactoglobulin, which is normally internalized and degraded by PT endolysosomes^2,3^, was dramatically reduced in pronephric tubules of *ctns*^*del8/del8*^ compared with *ctns*^*+/+*^ zebrafish (Fig. 3e).

**Fig. 4:**
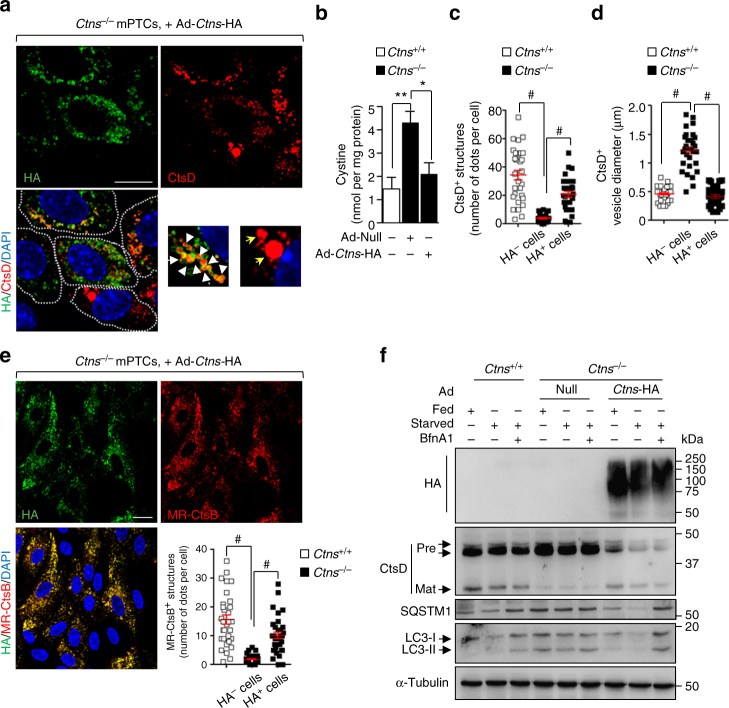
Rescue of lysosome–autophagy pathway by re-expressing CTNS in *Ctns*^−/−^ cells. **a**–**f** *Ctns*^−/−^ mPTCs were transduced with either Null (Ad-Null) or hemagglutinin-tagged *Ctns* (Ad-*Ctns*-HA) bearing adenoviral particles for 2 days. **a** Cells were immunostained with anti-HA (green) and anti-CtsD (red) antibodies, and analysed by confocal microscopy. Insets: high magnification of *Ctns-*HA^+^ vesicles containing CtsD. **b** Intracellular cystine levels were measured by HPLC. Two-tailed unpaired Student’s *t*-test, **P < 0.01 relative to *Ctns*^*+/+*^ mPTCs; *P < 0.05 relative to *Ctns*^−/−^ mPTCs transduced with Ad-Null. **c** Quantification of changes in the number of CtsD^+^ and **d** in the average vesicle size in cells from **a** (*n* = 30 cells pooled from three *Ctns* kidneys per condition; each point representing the number or the average size of CtsD^+^ vesicles in a cell; one-way ANOVA followed by Bonferroni’s post hoc test, ^#^*P* < 0.0001 relative to *Ctns*^*+/+*^ mPTCs or to HA^−^ *Ctns*^−/−^ mPTCs. **e** *Ctns* mPTCs were loaded with MR-CtsB peptide (1 μM, for 1 h at 37 °C) and immunostained with anti-HA antibody. Quantification of numbers of MR-CtsB^+^ structures (*n* = 33 cells pooled from three *Ctns* kidneys per condition, each point representing the number of MR-CtsB^+^ vesicles in a cell; one-way ANOVA followed by Bonferroni *post hoc* test, ^#^*P* < 0.0001 relative to *Ctns*^*+/+*^ mPTCs or to HA^*−*^ *Ctns*^−/−^ mPTCs). **f** *Ctns* mPTCs were cultured in fed or starved medium in presence or in absence of 250 nM BfnA1 for 4 h. The cells were lysed and subjected to western blotting analysis for the protein levels of HA, CtsD, SQSTM1, and LC3. α-Tubulin was used as a loading control, *n* = 3 independent experiments. Plotted data represent mean ± SEM. The nuclei are counterstained with DAPI (blue). Yellow indicates colocalization. Scale bars are 10 μm

The key role of cystinosin depletion in lysosomal–autophagy dysfunctions was assessed by transducing *Ctns*^−/−^ cells with an adenovirus that expresses mouse hemagglutinin (HA)-tagged cystinosin (Ad-*Ctns*-HA; Fig. 4). The functional re-expression of HA-CTNS protein at late endosomal/lysosomal compartments in *Ctns*^−/−^ cells (Fig. 4a; Supplementary Fig. 6) lowered the intracellular cystine content (Fig. 4b), rescued the lysosomal dynamics (Fig. 4c, d), activated cathepsin-B within lysosomes (Fig. 4e), and augmented the lysosome-mediated degradation of autophagy substrates SQSTM1 and LC3-II (Fig. 4f) when compared with *Ctns*^−/−^ cells transduced with an empty vector. These features were abolished by treating the HA-CTNS expressing *Ctns*^−/−^ cells with BfnA1 (Fig. 4f), indicating that cystinosin deficiency blocks autophagosome clearance by compromising lysosome function.

### Disruption of autophagy causes epithelial cell dysfunction

As autophagy mediates cellular homeostasis, we tested whether and how its disruption may cause epithelial dysfunction. Inactivation of basal autophagy was obtained by transducing mPTCs with an adenovirus expressing a short hairpin RNA (Ad-shRNA) against *Atg7* or by inhibiting the autophagy Beclin1/Vps-34 complex with SAR-405 or Spautin-1.

**Fig. 5:**
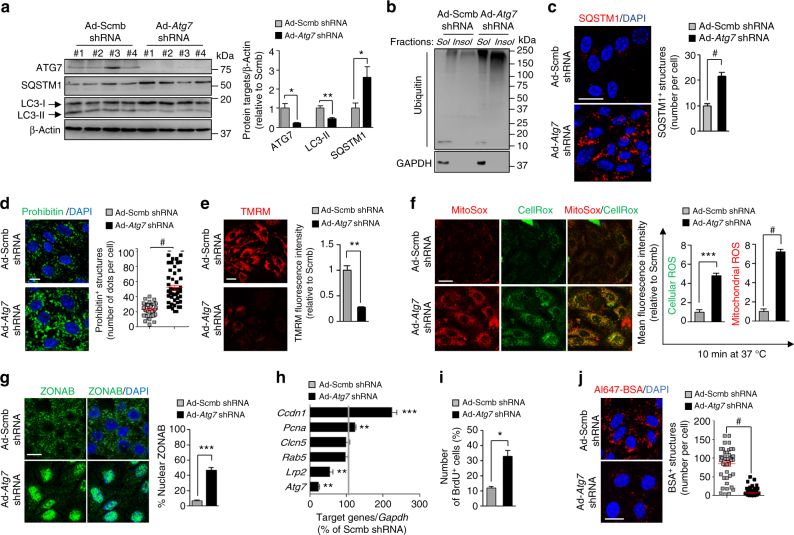
Failure of autophagy causes proliferation and dedifferentiation of PT cells. **a**–**j** mPTCs were transduced with either scrambled (Scmb) or *Atg7* adenoviral shRNAs for 5 days. **a** Western blotting and densitometric analyses of ATG7, SQSTM1, and LC3 levels. β-Actin was used as a loading control, *n* = 4 independent experiments. **b** Representative western blotting of the soluble and insoluble fractions derived from mPTCs and immunoblotted for ubiquitin and GAPDH; *n* = 3 independent experiments. **c** Confocal analysis of SQSTM1^+^ structures (red) in mPTCs (*n* = 143 cells pooled from four *Ctns* kidneys per condition) and **d** of prohibitin^+^ structures (green) in mPTCs (*n* = 40–50 cells pooled from three mouse kidneys per condition). **e** The cells were loaded with tetramethylrhodamine methyl ester (TMRM; mitochondrial membrane potential fluorescent probe, 50 nM for 30 min at 37 °C) and analysed by confocal microscopy. Quantification of TMRM fluorescence intensity obtained from five randomly selected fields per condition, with each containing ~ 20–25 cells. **f** mPTCs were loaded with CellROX (green, cellular ROS probe; 5 µM for 10 min at 37 °C) and Mito SOX (red, mitochondrial ROS probe; 2.5 µM for 10 min at 37 °C) analysed by confocal microscopy. Quantification of CellROX or MitoSOX fluorescence intensity obtained from three randomly selected fields per condition, with each containing ~ 20–25 cells. **g** mPTCs were immunostained with anti-ZONAB (green) antibody and analysed by confocal microscopy. Quantification of ZONAB^+^ nuclei (in percentage of the total nuclei) obtained from five randomly selected fields per condition, with each containing ~ 20–25 cells. **h** The mRNA levels of *Atg7*, *Clcn5*, *Rab5*, *Lrp2*, *Ccdn1*, and *Pcna* were analysed by real-time PCR (*n* = 4 independent experiments). **i** mPTCs were loaded with bromodeoxyuridine (BrdU, 1.5 μg ml^−1^ for 16 h at 37 °C), immunostained with anti-BrdU antibody and analysed by confocal microscopy. Quantification of numbers of BrdU^+^ cells (in percentage of total nuclei) obtained from five randomly selected fields per condition, with each containing ~ 20–25 cells. **j** The cells were loaded with Al647-BSA (50 μg ml^−1^ for 15 min at 37 °C) and imaged by confocal microscopy. Quantification of numbers of Al647-BSA^+^ structures (*n* = 49–85 cells pooled from three mouse kidneys per condition; each point representing the number of BSA^+^ structures in a cell). Plotted data represent mean ± SEM. Two-tailed paired Student’s *t*-test, *P < 0.05, **P < 0.01, ***P < 0.001, and ^#^*P* < 0.0001 relative to mPTCs transduced with Scmb shRNAs; NS, not significant. Nuclei are counterstained with DAPI (blue). Scale bars are 10 μm

We confirmed that either shRNA-mediated knockdown of *Atg7* (Fig. 5a) or autophagy inhibitors (Supplementary Fig. 7a–b) prevented the conversion of LC3-I to LC3-II and increased the levels of the SQSTM1 protein, and of aggregate-forming SQSTM1 and ubiquitin-positive inclusions (Fig. 5a–c and Supplementary Fig. 7c) in mPTCs. Disruption of autophagy led to the accumulation of dysfunctional mitochondria, as shown by increased levels of prohibitin (an inner mitochondrial membrane protein; Fig. 5d) and decreased resting mitochondrial membrane potential (Δψ_m_, assessed by quantitative confocal imaging of tetramethylrhodamine methyl ester (TMRM) dye; Fig. 5e and Supplementary Fig. 7d), and the induction of a major mitochondrial oxidative stress, as scored by elevated mtROS levels (MitoSOX; Fig. 5f and Supplementary Fig. 7e).

Importantly, either the genetic or the pharmacological impairment of autophagy induced the nuclear translocation of tight-junction ZO-1-associated Y-box factor ZONAB (Fig. 5g) and its activity (as measured by the regulation of the *Ccdn1*, *Pcna*, and *Lrp2* targets^27,28^ (Fig. 5h and Supplementary Fig. 7f). These changes were reflected by a phenotype switch that included abnormal cell proliferation, as evidenced by bromodeoxyuridine (BrdU) incorporation (Fig. 5i and Supplementary Fig. 7g), and dedifferentiation, as testified by reduced expression of the apical endocytic receptor megalin (Fig. 5h and Supplementary Fig. 7f) and by decreased endocytic uptake (Fig. 5j and Supplementary Fig. 7h). Collectively, these data demonstrate that the maintenance of mitochondrial function and homeostasis by autophagy is required for the terminal differentiation, hence regulating the reabsorptive capacity of PT epithelial cells.

### Dysfunctional mitochondria drive oxidative stress in *Ctns*^−/−^ cells

Having established that disruption of autophagy recapitulates the phenotype switch of proliferation dedifferentiation, we investigated how defective lysosomal degradation of autophagy substrates might lead to epithelial dysfunction in cystinosis.

**Fig. 6:**
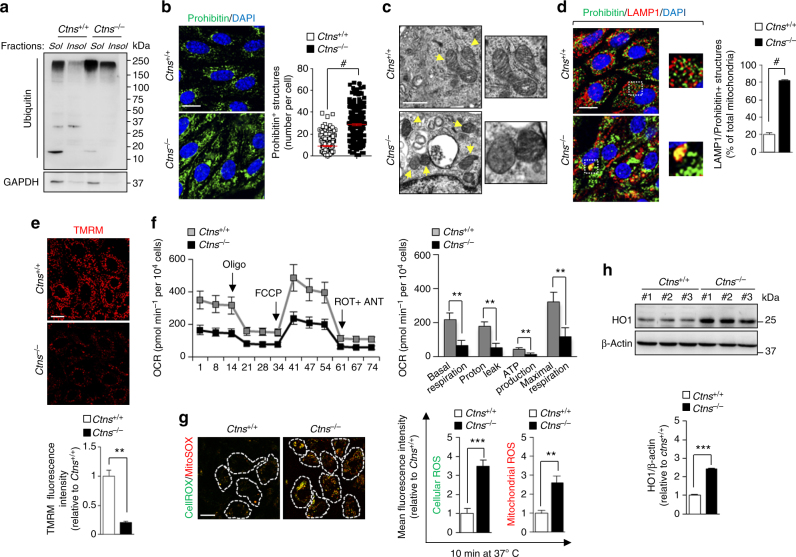
Accumulation of dysfunctional ROS-producing mitochondria in *Ctns*^*−/−*^ cells. **a** Representative western blotting of the soluble and insoluble fractions derived from *Ctns* mPTCs were immunoblotted for ubiquitin and GAPDH, *n* = 3 independent experiments. **b** Confocal analysis of prohibitin^+^ structures (green) in *Ctns* mPTCs (*n* = 240–280 cells pooled from three mouse kidneys per condition; each point representing the number of prohibitin^+^ structures in a cell). **c** Representative electron micrographs of mitochondria in *Ctns* mPTCs. Insets: mitochondria at higher magnification. **d** *Ctns* mPTCs were immunostained with anti-prohibitin (green) and anti-LAMP1 (red), and the numbers of LAMP1/prohibitin^+^ structures were quantified by confocal microscopy (percentage of total lysosomes; *n* = 5 randomly selected fields per condition, each containing ~ 20–25 cells). **e** *Ctns* mPTCs were loaded with tetramethylrhodamine methyl ester (TMRM; mitochondrial membrane potential fluorescent probe, 50 nM for 30 min at 37 °C) and analysed by confocal microscopy. Quantification of TMRM fluorescence intensity obtained from five randomly selected fields per condition, with each containing ~ 20–25 cells. **f** Oxygen consumption rate (OCR) and individual parameters for basal respiration, ATP production, proton leak, and maximal respiration. Oxygen consumption rates (OCRs) were measured under basal level and after the sequential addition of oligomycin (Oligo, 1 μM), FCCP (0.5 μM), and Rotenone (ROT; 1 μM) + Antimycin A (ANT; 1 μM); *n* = 3 independent experiments. **g** *Ctns* mPTCs were loaded with CellROX (cellular ROS probe; 5 μM for 10 min at 37 °C) and MitoSOX (mitochondrial ROS probe; 2.5 μM for 10 min at 37 °C) and analysed by live confocal microscopy. Quantification of CellROX or MitoSOX fluorescence intensity was obtained from five randomly selected fields per condition, with each containing ~ 20–25 cells. **h** Western blotting and densitometric analyses of HO1 levels. β-Actin was used as a loading control, *n* = 3 independent experiments. Plotted data are mean ± SEM. Two-tailed unpaired Student’s *t*-test **P < 0.01, ***P < 0.001, and ^#^*p* < 0.0001 relative to *Ctns*^*+/+*^ mPTCs. Nuclei are counterstained with DAPI (blue). Scale bars are 10 μm in **b**, **d**, **e**, and **g**; 1 μm in **c**

As autophagy delivers protein aggregates and organelles to endolysosome for cellular degradation, we analyzed the ability of *Ctns*^*−/−*^ mPTCs to clear polyubiquitinated aggregates and compromised mitochondria. Correlating with the stalled autophagy flux, we found an accumulation of ubiquitin-forming aggregates and abnormal mitochondria with disorganized cristae in *Ctns*^*−/−*^ compared with *Ctns*^+/+^ cells (Fig. 6a–c). The defective mitochondria in *Ctns*^*−/−*^ cells were retained within enlarged, dysfunctional lysosomes (Fig. 6d) and showed a major decrease in Δψ_m_ (Fig. 6e). Seahorse metabolic flux analyses measuring the overall consumption rates (OCRs) confirmed markedly decreased mitochondrial bioenergetics (baseline respiration, ATP turnover, and total respiratory capacity) in *Ctns*^*−/−*^ compared with *Ctns*^*+/+*^ mPTCs (Fig. 6f). These changes were paralleled by a major mitochondrial oxidative stress, as scored by the elevated mtROS levels (MitoSOX; Fig. 6g) and increased anti-oxidant response (heme oxygenase-1 (HO1); Fig. 6h). Overall, these findings indicate that impaired lysosome-mediated autophagic degradation of dysfunctional mitochondria causes a major oxidative stress in cystinosin-deficient cells.

### Abnormal tight junction signaling leads to epithelial dysfunction

Oxidative stress has been shown to induce the phosphorylation of the zona occludens-1 (ZO-1) adaptor protein, disrupting the integrity of the tight junction complex^29^. As ZO-1 traps the Y-box transcription factor ZONAB that is known to regulate the differentiation of PT cells^27,28^, an overproduction of ROS by dysfunctional mitochondria might alter tight junction integrity and trigger ZONAB-mediated signaling.

**Fig. 7:**
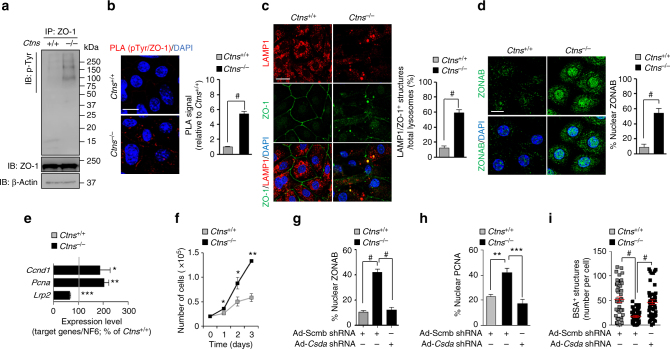
Augmented phosphorylation rate of ZO-1 promotes epithelial dysfunction in *Ctns*^*−/−*^ cells. **a** Tight junction ZO-1 protein was immunoprecipitated from *Ctns*^*+/+*^ and *Ctns*^*−/−*^ mPTCs, and its phosphorylation rate was examined by western blotting, *n* = 2 independent experiments. **b** Confocal analysis of endogenous phosphorylation of ZO-1 by proximity ligation assay (PLA), and **c** of ZO-1/LAMP1^+^ structures (in percentage of the total lysosomes) and **d** of ZONAB^+^ nuclei (in percentage of the total nuclei) in *Ctns* mPTCs. Quantifications in **b**, **c**, and **d** were obtained from five randomly selected fields per condition, with each containing ~ 20–25 cells; two-tailed unpaired Student’s *t*-test, ^#^*P* < 0.0001 relative to *Ctns*^*+/+*^ mPTCs. **e** The mRNA levels of *Lrp2, Ccdn1*, and* Pcna* in *Ctns* microdissected proximal tubules were analysed by real-time PCR, *n* = 3 mice per group. Two-tailed unpaired Student’s *t*-test, *P < 0.05, **P < 0.01, and ***P < 0.001 relative to *Ctns*^*+/+*^ microdissected proximal tubules. **f** Growth curves of *Ctns* mPTCs. Two-tailed unpaired Student’s *t*-test, *P < 0.05 and **P < 0.01 relative to *Ctns*^*+/+*^ mPTCs, *n* = 3 independent experiments. **g**–**i** *Ctns* mPTCs were transduced with Scmb or *Csda* adenoviral shRNAs for 5 days. **g** Confocal analysis and quantification of ZONAB^+^ nuclei and **h** of PCNA^+^ nuclei in *Ctns* mPTCs. The quantifications (expressed in percentage of the total nuclei) were obtained in **g** and **h** from four randomly selected fields per condition, with each containing ~ 20–25 cells. **i** The cells were loaded with Al647-BSA (50 μg ml^−1^ for 15 min at 37 °C) and imaged by confocal microscopy. Quantification of numbers of Al647-BSA^+^ structures (*n* = 45–50 cells pooled from three mouse kidneys per condition; each point representing the number of BSA^+^ structures in a cell). One-way ANOVA followed by Bonferroni’s post hoc test, **P < 0.01, ***P < 0.001, and ^#^*P* < 0.0001 relative to *Ctns*^*+/+*^ mPTCs transduced with Ad-Scmb shRNA or to *Ctns*^*−/−*^ mPTCs transduced with Ad-Scmb shRNA. Plotted data are mean ± SEM. Nuclei counterstained with DAPI (blue). Scale bars are 10 μm

To test this hypothesis, we immunoprecipitated ZO-1 from mPTCs and noted that it was more heavily phosphorylated in *Ctns*^*−/−*^ vs. *Ctns*^*+/+*^ cells (Fig. 7a). This result was supported by the proximity ligation assay (PLA) revealing an increased cytoplasmic phosphorylation of ZO-1 in *Ctns*^*−/−*^ cells (Fig. 7b). ZO-1, which is normally located at cell membrane boundaries as shown in *Ctns*^*+/+*^ cells, was found to be misrouted and trapped within LAMP1-positive compartments in *Ctns*^*−/−*^ cells (Fig. 7c). Consistent with the loss of integrity of tight junctions, there was a nuclear translocation of ZONAB (Fig. 7d) with subsequent increase in its transcriptional targets *Ccdn1*, *Pcna*, and *Lrp2* (Fig. 7e). These changes in *Ctns*^*−/−*^ cells were coupled to increased proliferation, as evidenced by measuring cell growth curves (Fig. 7f) or by the nuclear enrichment of PCNA (Fig. 7h and Supplementary Fig. 1e), contrasting with decreased receptor-mediated endocytosis of albumin (Fig. 7i and Supplementary Fig. 1f).

The link between defective autophagy, compromised mitochondria, and oxidative stress was substantiated by the increased phosphorylation rate of ZO-1 induced by the shRNA-mediated knockdown of *Atg*7 in mPTCs (Supplementary Fig. 8a) or by the treatment of the cells with autophagy inhibitors (SAR-405 and Spautin-1; Supplementary Fig. 8b), with H_2_O_2_ (Supplementary Fig. 8c) or with the mitochondrial complex I inhibitor Rotenone (ROT)-stimulating mitochondrial ROS (Supplementary Fig. 8d,e). Treatment with ROT induced ZONAB translocation and signaling (Supplementary Fig. 8f, g), reflected by increased proliferation (Supplementary Fig. 8h) and decreased endocytic uptake (Supplementary Fig. 8i).

The relevance of the abnormal tight junction signaling in cystinosis was confirmed by gain- and loss-of-function experiments in mPTCs. shRNA-mediated knockdown of *Csda* encoding ZONAB (Fig. 7g–i and Supplementary Fig. 9a–c) or overexpression of exogenous *TJP1* encoding ZO-1 (Supplementary Fig. 9d, e), which functionally inhibits ZONAB^27,28^, significantly decreased the proliferation markers and rescued the endocytic uptake in *Ctns*^*−/−*^ cells. Conversely, depletion of ZO-1 (shRNA-mediated knockdown of *Tjp1*; Supplementary Fig. 10a–c) or overexpression of *Csda* (Supplementary Fig. 10d) triggered the activation of ZONAB signaling (Supplementary Fig. 10e) with increased cell proliferation and decreased endocytic uptake of albumin (Supplementary Fig. 10f) in wild-type cells.

### Mitochondrial ROS disrupt tight junctions through Gα12 /Src activation

Finally, we investigated how aberrant mitochondria-producing ROS disrupt tight junctions in cystinosis epithelial cells. Given the accumulation of abnormal mitochondria-producing ROS (Fig. 6) and higher phosphorylation rate of ZO-1 (Fig. 7) in *Ctns*^*−/−*^ PT cells, we hypothesized that high levels of mitochondrial ROS may stimulate the Gα12 protein that triggers the disruption of tight junctions through Src-mediated phosphorylation of ZO-1^29–31^. This would in turn activate ZONAB signaling and drives the epithelial dysfunction.

**Fig. 8:**
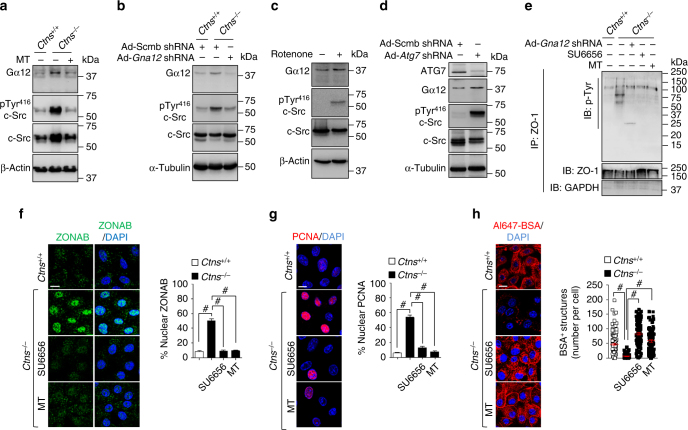
Mitochondrial ROS triggers Ga12/Src-mediated destabilization of ZO-1. **a** Representative western blotting of Gα12, pTyr^416^ c-Src, and c-Src protein levels in *Ctns* mPTCs treated with or without the mitochondria-targeted antioxidant Mito-TEMPO (MT, 10 μM for 24 h). β-Actin was used as a loading control, *n* = 3 independent experiments. **b** Representative western blotting of Gα12, pTyr^416^ c-Src, and c-Src protein levels in *Ctns* mPTC transduced with either Scmb or *Gna12* adenoviral shRNAs for 5 days. α-Tubulin used as a loading control, *n* = 2 independent experiments. **c** Representative western blotting of Gα12, pTyr^416^ c-Src, and c-Src protein levels in mPTCs treated with Rotenone (250 nM for 8 h) or **d** transduced with Scmb or *Atg7* adenoviral shRNAs for 5 days. In **c** and **d**, β-Actin and α-Tubulin were used as a loading control, *n* = 3 independent experiments. **e** Tight junction ZO-1 protein was immunoprecipitated from *Ctns*^*+/+*^ or *Ctns*^*−/−*^ mPTCs and from *Ctns*^*−/−*^ mPTCs transduced with Ad-*Gna12* shRNA or treated with either mitochondria-targeted antioxidant Mito-Tempo (MT, 10 μM for 24 h) or with a selective Src-kinase inhibitor SU6656 (5 μM for 24 h) and the phosphorylation rate was examined by western blotting. **f**–**h** *Ctns* mPTCs were treated with either mitochondria-targeted antioxidant MitoTempo (MT, 10 μM for 24 h) or a selective Src-kinase inhibitor SU6656 (5 μM for 24 h). **f** *Ctns* mPTCs were immunostained with anti-ZONAB (green) antibody and imaged by confocal microscopy. Quantification of ZONAB^+^ nuclei (in percentage of the total nuclei) was obtained from five randomly selected fields per condition, with each containing ~ 20–25 cells. **g** *Ctns* mPTCs were immunostained with anti-PCNA antibody and imaged by confocal microscopy. Quantification of PCNA^+^ nuclei (in percentage of the total nuclei) obtained from five randomly selected fields per condition, with each containing ~ 20–25 cells. **h** The cells were loaded with Al647-BSA (50 μg ml^−1^ for 15 min at 37 °C) and imaged by confocal microscopy. Quantification of numbers of Al647-BSA^+^ structures (*n* = 100 cells pooled from three mouse kidneys per condition; each point representing the number of BSA^+^ structures in a cell). One-way ANOVA followed by Bonferroni’s post hoc test, ^#^*P* < 0.0001 relative to *Ctns*^*+/+*^ mPTCs or to untreated *Ctns*^*−/−*^ mPTCs. Plotted data are mean ± SEM. The nuclei are counterstained with DAPI (blue). The scale bars are 10 μm

Consistent with this hypothesis, mPTCs from *Ctns*^*−/−*^ kidneys (Fig. 8a, b) displayed an increased protein abundance of Gα12 and activated Src tyrosine kinase (monitored by the increased phosphorylation of pTyr^416^-Src; Fig. 8a, b). Likewise, wild-type mPTCs treated with ROT (Fig. 8c) or accumulating aberrant mitochondria-producing ROS (shRNA-mediated knockdown of *Atg7*; Fig. 8d) also exhibited an abnormal activation of Gα12/Src. This activation paralleled the increased phosphorylation of ZO-1, tight junction disruption, and epithelial dysfunction described above (Fig. 5 and Supplementary Fig. 8).

Having verified that the high level of mitochondrial ROS drives epithelial dysfunction by activating Gα12/Src kinase pathway, we tested whether the neutralization of mitochondrial ROS/Gα12/Src signaling loop may avert the loss of tight junction integrity and rescue the function of cystinosis cells. We incubated *Ctns*^*−/−*^ mPTCs with mitochondria-localized-oxygen scavenger Mito-TEMPO (MT, 10 μM for 24 h; Supplementary Fig. 11a, b^32^) or with a selective c-Src family kinase inhibitor SU6656 (5 μM for 24 h^33^), or transduced the cells with Ad-shRNA against *Gna12*.

The blockage of the mitochondrial ROS/Gα12/Src signaling cascade by these pharmacologic or genetic interventions reversed the abnormal activation of Gα12/Src (Fig. 8a, b), preventing the phosphorylation of ZO-1 (Fig. 8e and Supplementary Fig. 11c) and its lysosomal accumulation, which increased ZO-1 abundance at cell boundaries (Supplementary Fig. 11d). In turn, these changes inhibited the nuclear translocation of ZONAB (Fig. 8f) and restored the differentiation and endocytic uptake of albumin in cystinosis cells (Fig. 8g, h). Of note, the rescue induced by MT treatment was prevented by overexpressing *Csda*-HA in *Ctns*^*−/−*^ mPTCs (Supplementary Fig. 12a, b), supporting the role of ZONAB.

Taken together, these data suggest that the excess of mitochondrial ROS, resulting from impaired lysosome-mediated autophagic degradation of damaged mitochondria, triggers Gα12/Src-mediated phosphorylation of ZO-1, which disrupts tight junction integrity and activates ZONAB signaling, causing epithelial dysfunction in cystinosis cells.

### Neutralizing mitochondrial ROS improves epithelial function in cystinosis

**Fig. 9:**
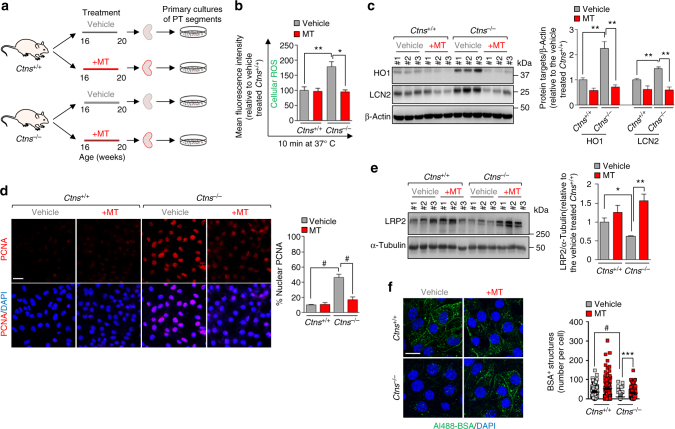
Mitochondrial ROS scavenging ameliorates PT cell function in *Ctns*^*−/−*^ mice. **a** *Ctns* mice were treated for 30 days with daily intraperitoneal injections of either saline or MT (1 mg kg^−1^ body^-^weight, *n* = 4 mice for group). Following treatment, mPTCs obtained from microdissected *Ctns* kidneys were analysed. **b** The mPTCs were loaded with CellROX (5 μM for 10 min at 37 °C) and analysed by confocal microscopy. Quantification of fluorescence intensity was obtained from five randomly selected fields per condition, with each containing ~ 20–25 cells. **c** Western blotting and densitometric analyses of HO1 and lipocalin-2 (LCN2) protein levels. β-Actin was used as a loading control. **d** The cells were immunostained for PCNA and imaged by confocal microscopy. Quantification of PCNA^+^ nuclei (in percentage of the total nuclei) obtained from five randomly selected fields per condition, with each containing ~ 20–25 cells. **e** Western blotting and densitometric analyses of LRP2 protein levels. α-Tubulin was used as loading control. **f** The cells were loaded with Al488-BSA (50 μg ml^−1^ for 15 min at 37 °C), fixed, and analyzed by confocal microscopy. Quantification of numbers of Al488-BSA^+^ structures (*n* = 100 cells pooled from three mouse kidneys per condition; each point representing the number of BSA^+^ structures in a cell). Nuclei counterstained with DAPI (blue) in **d** and **f**. Plotted data show mean ± SEM. One-way ANOVA followed by Bonferroni’s post hoc test, *P < 0.05, **P < 0.01, ***P < 0.001, and ^#^*P* < 0.0001 relative to mPTCs from vehicle-treated *Ctns*^*+/+*^ or *Ctns*^*−/−*^ mice. Scale bars are 10 μm

To enhance the translational potential of these findings, we administered MT (1 mg kg^−1^ body weight) to 16-week-old *Ctns* mice by daily intraperitoneal injections (Fig. 9a). After 30 days of treatment, mPTCs were isolated from the *Ctns*^*−/−*^ kidneys to score the epithelial function by measuring the endocytic uptake of albumin. Treatment with MT effectively reduced ROS production and oxidative stress (Fig. 9b, c), lowered levels of the kidney injury marker lipocalin-2 (LCN2^34^) (Fig. 9c), abrogated abnormal cell proliferation (Fig. 9d) and restored abundance of endocytic receptor megalin (LRP2; Fig. 9e), reflected by a significant recovery of the ligand uptake (Fig. 9f) in *Ctns*^*−/−*^ cells. Thus, modulation of mitochondrial oxidative stress ameliorates epithelial function in nephropathic cystinosis.

## Discussion

**Fig. 10:**
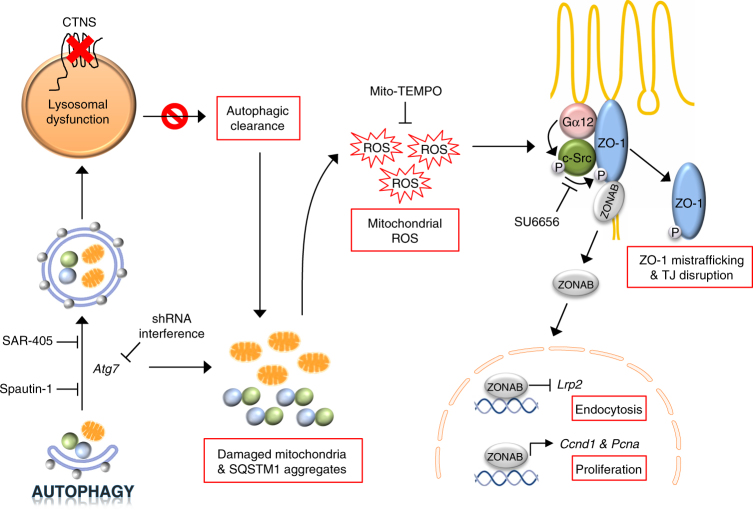
A working model depicting the link between defective lysosome–autophagy pathways and epithelial dysfunction in cystinosis. The black arrows identify the sequence of events occurring within cystinosis PT cells. The loss of the cystinosin (CTNS) transport system leads to lysosomal dysfunction. This impairs the cellular clearance of autophagosomes containing SQSTM1^+^ aggregates and/or damaged mitochondria. Similarly, pharmacological and genetically mediated failure of autophagy (with SAR-405 and Spautin-1 inhibitors or with shRNA-mediated knockdown of *Atg7*) increases the SQSTM1^+^ aggregates and/or damaged mitochondria-producing ROS. The mitochondrial oxidative stress enables the Gα12/Src-mediated phosphorylation of the tight junction adapter protein ZO-1, resulting in its misrouting to endolysosomal compartment. The disruption of tight junction integrity releases the ZO-1-associated Y-box factor ZONAB, which promotes cell proliferation and represses apical endocytic receptors such as LRP2, causing epithelial dysfunction in PT cells. Conversely, neutralization of excessive mitochondrial oxidative stress (with mitochondria-targeted oxygen scavenger Mito-TEMPO) or blockade of the Gα12/Src regulatory loop (with a selective Src-kinase inhibitor Su6656) restores the differentiation and transport properties in cystinosis PT cells

The endolysosomal system regulates the transport activity of specialized epithelial cells, sustaining their role in homeostasis. Congenital defects in lysosomal transporters, as exemplified by cystinosis, cause PT dysfunction and RFS. By combining genetic and pharmacologic approaches in vitro and in vivo, we decipher the link between lysosomal disease and epithelial dysfunction in cystinosis. We demonstrate that lysosomal dysfunction results in defective autophagy-mediated clearance of damaged mitochondria-producing ROS, disruption of tight junction integrity, and activation of a signaling pathway causing epithelial cell proliferation and dedifferentiation, with loss of reabsorptive capacity (Fig. 10). These data reveal the fundamental importance of the lysosome-mediated autophagic clearance in maintaining epithelial differentiation and offer novel therapeutic perspectives to restore epithelial transport capacity downstream of the primary lysosomal defect.

Cystinosin deficiency induces a major alteration in lysosomal dynamics^9,35,36^, paralleled by increased numbers of autophagosomes in the PT cells of *Ctns*^*−/−*^ mice. Similarly, abnormal lysosomes and LC3-marking autophagosomes heighten in cystine-accumulating pronephric tubules in *ctns*-deficient zebrafish, demonstrating the evolutionary conservation of this connection across vertebrates. Contrasting with cystine storage and defective lysosome–autophagy pathways, the *ctns* zebrafish larvae appear morphologically normal and display neither obvious development defects nor signs of PT dysfunction. As cystinosis is typically a storage disease, longitudinal studies are needed to provide a more comprehensive characterization of the phenotype after 5 days post fertilization (dpf) in this model.

The accumulation of the autophagy substrate SQSTM1, which is normally degraded within autolysosomes, suggests an abnormal autophagy flux in cystinosin-deficient PT cells, in line with recent observations stating an impairment of autophagy flux in many lysosome storage diseases^37,38^ and evidence in human cells and kidney biopsies from cystinosis patients^16^. Evidence supporting the failure to degrade autophagy cargoes in primary PT *Ctns*^*−/−*^ cells include the following: abnormally elevated numbers of mature autophagosomes under normal growth conditions; failure to clear AVs formed after starvation-induced autophagy, mimicking BfnA1 action; inability of BfnA1 to further induce the LC3-II protein levels; and impaired degradation of the resting AVs by a selective PI3K3/Vps34 inhibitor. In contrast, upstream events regulating autophagosome biogenesis including Atg7–Atg12 conjugation and phagophore formation were not affected. Altogether, these data indicate that the functional loss of cystinosin, causing lysosomal cystine storage, delays the autophagy flux, causing an accumulation of autophagosomes in vitro and in vivo. Of note, the autophagy flux was found to be fully functional in fibroblasts derived from *Ctns* mice^39^, in contrast with the predictions based on the defective mTORC1 signaling in cystinosis cells^17^. The reason for these discrepancies would require further studies to understand the tissue and cell-specific effects of cystinosin depletion.

The question remained how the lysosomal defect could impair autophagy flux and lead to epithelial dysfunction. A blockage of the degradation of autophagic material may occur at any step after autophagosome formation, due to factors regulating the trafficking of autophagosomes to lysosomes or the fusion between both compartments. For instance, accumulation of LC3-positive organelles unable to fuse with lysosomes has been observed in many LSDs^40^. Accordingly, a potential cause for the impaired autophagy flux observed in cystinosis could be a delayed fusion between lysosomes and autophagosomes. However, when treating *Ctns*^*−/−*^ cells with short incubations of BfnA1, which accurately measure the autophagy flux^41^, we observed a substantial co-localization of LC3-positive vesicles with the lysosome marker LAMP1, suggesting that the delivery and autophagosome–lysosome fusion is not compromised in cystinotic cells. Another indication is the perinuclear lysosomal clustering in *Ctns*^*−/−*^ cells; such centripetal movement of lysosomes has been shown to mediate the autophagic degradation of biomacromolecules^20,42^.

As the autophagy flux also relies on the degradative capacity of lysosomes^11^, an impaired lysosomal function may explain the accumulation of autophagosomes in cystinosis cells. We postulated that cystinosin deficiency may affect lysosome function either by controlling the delivery of newly synthesized lysosomal cathepsins from Golgi to the lysosome or by inhibiting the processing/maturation of cathepsins within endolysosome compartment. Our results support the latter hypothesis, evidencing an impairment of lysosome proteolysis (observed both in vitro and in vivo) as a result of defective cathepsin activation despite an efficient delivery of newly synthesized cathepsins from Golgi to lysosomes. Furthermore, the rescue of lysosomal cystine homeostasis through the transient expression of cystinosin in *Ctns*^*−/−*^ cells resulted in multi-level reactivation of the autophagy–lysosome degradative pathways. Taken together, these findings support a role of cystinosin—beyond its function in cystine transport—in maintaining the lysosomal response to the arrival of the autophagy cargoes, hence in cellular homeostasis.

The conjugation of impaired lysosomal dynamics and altered lysosomal degradative capacity is strikingly similar to the cellular changes resulting from the accumulation of monoclonal light chains (κLCs) within endolysosomes of PT cells, causing a similar epithelial dysfunction^22^. Furthermore, the uncontrolled increase in lysosomal PtdIns(4,5)P_2_ that results from the loss-of-function of the PtdIns(4,5)P_2_ 5-phosphatase oculo cerebro renal Lowe syndrome (OCRL), leads to lysosomal dysfunction and autophagosome accumulation in PT cells from patients with Lowe syndrome, another congenital disorder causing PT dysfunction and RFS^43^. These data suggest that lysosomal accumulation of cystine or specific κLCs or PtdIns(4,5)P_2_ may have similar functional consequences on the epithelial phenotype, emphasizing the role of endolysosomes as crucial signaling hub to ensure cellular homeostasis. As lysosomal acidification is necessary for the activation of cathepsins, one could speculate that the vacuolar-type H^+^-ATPase (V-ATPase) complex might be altered in absence of cystinosin^44^. Potential factors affecting V-ATPase efficiency in LSD may include the following: storage of cholesterol in the endolysosome membranes^45^; luminal oxidation of lysosomal thiols by free cystine^10^; and accumulation of cystine sustaining mTORC1 activity and negatively regulating lysosome biogenesis by suppressing TFE/MiTF signaling^46,47^. Recent studies showing that cystinosin is a member of the lysosomal machinery that controls mTORC1 activity in vitro^17^, and that overexpression of transcription factor EB (TFEB) stimulates lysosomal cargo processing^48^ support the concept. Regardless of the mechanism, impaired cellular clearance leads to accumulation of autophagy substrates, such as dysfunctional mitochondria and ubiquitinated proteins. Although autophagy deficits have been reported in LSDs^37,38,49^, the impact of lysosome–autophagy defects on epithelial cell function remains unknown. Basal autophagy operates to preserve the integrity of subcellular compartments, including damaged mitochondria^50,51^. Lack of autophagy completion, due to impaired lysosomal degradation capacity or through inactivation of essential genes, leads to the persistence of ubiquitinated proteins (including p62/SQSTM1) and dysfunctional, ROS-producing mitochondria^38^. Our results demonstrate the importance of this cellular quality-control mechanism for the function of epithelial cells. Genetic or pharmacologic blockage of basal autophagy resulted in accumulation of ubiquitinated proteins and damaged/dysfunctional mitochondria, leading to abnormal cell proliferation and apical dedifferentiation reflected by decreased uptake capacities. Similar to the changes observed in autophagy-deficient cells, there was a remarkable accumulation of SQSTM1- and ubiquitin-forming aggregates in *Ctns*^*−/−*^ cells, with dysfunctional mitochondria accumulating within enlarged, non-degradative lysosomes and generating enhanced levels of mitochondrial ROS. These results indicate the importance of maintaining a healthy mitochondria repertoire to substantiate the solute transport activity in PT cells^52^. In fact, primary mitochondrial diseases cause epithelial transport defects and RFS^4,52^. Oculo Cerebro Renal Lowe syndrome

The link between abnormal mitochondrial autophagy and oxidative stress is well documented^53,54^, but how excessive mitochondrial ROS production may cause epithelial cell dysfunction? Highly specific junctions modulate the epithelial phenotype along the renal tubule segments. The levels of the tight junction adaptor protein ZO-1 and the localization of ZONAB, a Y-box transcription factor that interacts with ZO-1, modulate the switch from a proliferative to differentiated state in PT cells^28^. For instance, the absence of the endosomal channel CFTR^55^ leads to reduced ZO-1 stability, promoting nuclear translocation of ZONAB and cell proliferation^56^. Similarly, shRNA-mediated knockdown of *Tjp1* encoding ZO-1 in primary PT cells triggers an aberrant activation of tight junction-associated (ZONAB) signaling, with subsequent induction of a phenotype switch associating abnormal cell proliferation and dedifferentiation, mimicking the epithelial dysfunction associated with cystinosis. An abnormal activity of ZONAB has also been evidenced when κLCs accumulate within dysfunctional endolysosomes^22^. Furthermore, gain-of-function interventions targeting tight junction-associated ZONAB signaling rescue epithelial differentiation and reabsorptive capacity in cystinosis PT cells. Taken together, these data suggest that the maintenance of tight junction integrity acts an essential regulator of epithelial cell differentiation.

Recent advances have illuminated the understanding of regulatory mechanisms involved in tight junction architecture and remodeling^29–31^. In particular, changes in the cellular rate of Gα12/Src-mediated phosphorylation of ZO-1 associated with oxidative stress may disrupt tight junctions and cause cell damage^29–31^. In line with these observations, our results substantiate that the aberrant production of mitochondrial-ROS enhances the Gα12/Src-mediated phosphorylation of ZO-1 and its subsequent mistargeting to enlarged, non-degradative endolysosomes. In turn, the loss of tight junction integrity promotes ZONAB signaling and transcription of target genes that increase cell proliferation and decrease differentiation^27,28^, resulting in defective endocytosis in *Ctns*^*−/−*^ cells. The relevance of this abnormal cascade is supported by the gain- and loss-of-function approaches targeting Gα12 or ZO-1/ZONAB, or by pharmacological interventions impeding activation of Gα12/Src signaling axis in *Ctns*^*−/−*^ PT cells and also in *Ctns*^*+/+*^ PT cells under mitochondria-derived oxidative stress conditions (ROT or blockade of autophagy). Consistent with this, treatment of *Ctns*^*−/−*^ mice and their derived mPTCs with mitochondrial-targeted antioxidants, which are clinically tested in various mitochondrial diseases^57^, not only repaired dysfunctional mitochondria and averted mitochondrial oxidative stress, but also rescued the integrity of tight junctions and the differentiation and endocytic uptake capacity of the cells. These data extend the previous findings showing that swan-neck deformities of PT segments^58^ could be delayed in *Ctns*-knockout mice by administrating mitoquinone, an anti-oxidant compound that acts on mitochondria.

In conclusion, we identify a novel link between lysosomal storage and dysfunction, impaired autophagic clearance, and loss of transport capacity in epithelial cells. These findings substantiate the role of endolysosome system in preserving the autophagy-mediated quality control of mitochondria, which are crucial for the high transport activities performed by specialized epithelial cells. They also demonstrate the cross-talk between mitochondria and tight-junction associated signal transduction pathways regulating the epithelial phenotype. The effect of an antioxidant compound specifically targeting mitochondria on the function of *Ctns*^*−/−*^ cells provides a promising approach to alleviate the loss of vital metabolites in nephropathic cystinosis.

## Methods

### Antibodies and reagents

The following antibodies were used in this study: rat anti-LAMP1 (sc-19992, Santa Cruz Biotechnology; 1:500); rabbit anti-LC3(PM036, MBL; 1:100), rabbit anti-LC3 (NB100-2331, Novus Biologicals; 1:200), rabbit anti-p62/SQSTM1 (PM045, MBL; 1:200); rabbit anti-GAPDH (2118, Cell Signaling Technology; 1:10,000); mouse anti-β-actin (A2228, Sigma-Aldrich; 1:10,000); goat anti-CtsD (sc-6486, Santa Cruz Biotechnology; 1:500); rabbit anti-GM130 (ab52649, Abcam; 1:500); mouse anti-Ubiquitin (sc-8017, Santa Cruz Biotechnology; 1:1,000); mouse anti-PCNA (M0879, DAKO; 1:500); rabbit anti-Histone-3 (H3; ab4729, Abcam; 1:1,000); mouse anti-α-tubulin (T5168, Sigma-Aldrich; 1:1,000); rabbit anti-Prohibitin (ab28172, Abcam; 1:200); rabbit anti-ATG16L (D6D5) (8089, Cell Signaling; 1:400); rabbit anti-ATG7 (A2856, Sigma-Aldrich; 1:200); mouse anti-BECN1 (sc-48341, Santa Cruz Biotechnology; 1:500); rat anti-HA (11867423001, Roche; 1:500); rabbit anti-Calnexin (C4731, Sigma-Aldrich; 1:500); mouse anti-Phospho-Tyr-99 (PY99; sc-7020, Santa Cruz Biotechnology; 1:500); rabbit anti-ZO-1(sc-10804, Santa Cruz Biotechnology; 1:100); rabbit anti-ZONAB (A303-070A, Bethyl Laboratories; 1:100); mouse anti-Gα12 (sc-515610, Santa Cruz Biotechnology; 1:200); rabbit anti-phospho-Src Family (pTyr^416^; 6943, Cell Signaling; 1:500), rabbit anti-Src (2109, Cell Signaling; 1:500); mouse anti-PtdIns-3P (Z-P003, Echelon Biosciences; 1:200); rabbit anti-HO1 (ab13243, Abcam; 1:500); rabbit anti-LCN2 (ab63929, Abcam; 1:200); and goat anti-green fluorescent protein (GFP) (AB0020-500, SICGEN; 1:500). Sheep anti-megalin antibody (LRP2; 1:1,000) was kindly provided by P. Verroust and R. Kozyraki (INSERM, Paris, France). Compounds included BfnA1 (ALX-380-030, Enzo Life Sciences), PIK3C3/Vps34 inhibitor SAR-405 (A8883, APExBIO), autophagy inhibitor Spautin-1 (S7888, Selleckchem.com), hydrogen peroxide (H_2_O_2_; H1009 Sigma-Aldrich), ROT (R8875, Sigma-Aldrich), SU6656 (s9692, Sigma-Aldrich), and Mito-TEMPO (MT; ALX-430-150-M005, Enzo Life Sciences).

### Generation and maintenance of *ctns* zebrafish

*ctns*-specific left and right TALENs (*ctns*-TALENs) were constructed in according to Golden Gate TALEN assembly protocol and using the Golden Gate TALEN and TAL Effector Kit 2.0 (Addgene, Kit 1000000024). CIscript-GoldyTALEN was a gift from Daniel Carlson & Stephen Ekker (Addgene, plasmid 38142). TALENs were designed with the TAL Effector Nucleotide Targeter 2.0 software on the Website of Cornell University. The TALENs target the exon 3 of *ctns* zebrafish gene: left TALEN-F: 5′-TCTTTTAATCCTTTGTGTTCACA-3′ and right TALEN-R: 5′-CATCTGTAACGGTTTATTTCAAT-3′.

The spacer between two TALEN target sites is ~ 15 nucleotides and contains an AciI restriction site in the middle, which is used for mutant screening. The TALEN expression plasmids were linearized with BamHI and then used for in vitro transcription (mMESSAGE mMACHINE T3 kit, Ambion). Approximately 1 nl of TALEN mRNAs (400 ng µl) was injected into one-cell stage zebrafish (*Danio rerio*) embryos. After 24 h, genomic DNA was extracted from injected embryos with normal appearance. Targeted genomic loci were amplified by using primers designed to anneal ~ 240 base pairs and mutant allele was detected by AciI digestion of PCR product. The TALEN-injected embryos were raised to adulthood (F0) and outcrossed with wild-type zebrafish. The embryos were then raised to adulthood (F1) for screening of heterozygous carriers. We identified a heterozygous carrier harboring *ctns* ^*+/del8*^ mutation and (F1) generations were crossed for obtaining homozygous mutant carrying *ctns*^*del8/del8*^. Zebrafish were kept at day/night cycle of 14/10 h at 28 °C. Zebrafish embryos were obtained through natural spawning in the facility of the University of Zurich and raised in E3 medium containing 0.01% methylene blue and prepared for analyses. Adult zebrafish were maintained in the zebrafish facility of Hubrecht Institute (Utrecht, The Netherlands). Zebrafish were killed by immersion in system water containing 300 µg ml^−1^ tricaine methane sulfonate (MS222). The experiments performed on adult fish (Hubrecht Institute) were approved by the Animal Care and Use Committee of the Royal Netherlands Academy of Arts and Sciences.

### Rescue experiments in *ctns* zebrafish

The plasmids containing wild type or mutant (ΔDVVF346-349) human *CTNS* compleentary DNA were kindly provided by C. Antignac (INSERM, Paris, France). The T3 promoter sequence was cloned into the XhoI restriction site of the enhanced GFP vector by using forward primer: 5′-TCGAGGATCCATTAACCCTCACTAAAGGGAAC-3′ and reverse primer: 5′-TCGAGTTCCCTTTAGTGAGGGTTAATGGATCC-3′. Either wild type or mutant *CTNS* mRNA was synthesized by using the mMESSAGE mMACHINE T3 transcription Kit (Invitrogen). The microinjection of either wild type or mutant *CTNS* mRNA was performed in *ctns* ^*del8/del8*^ zebrafish embryos at one-cell stage and subsequently collected at 5 dpf for cystine measurements. Where indicated, a working solution of cysteamine at 1 mM was prepared by diluting the stock solution within E3 medium. After dechorionation, *ctns* zebrafish larvae at 2 dpf were incubated in the E3 medium in the presence or in the absence of cysteamine until to the sampled day (5 dpf), and the zebrafish medium was renewed daily. Twenty zebrafish embryos per group were pooled and homogenized by sonication, and prepared for cystine measurements.

### Mouse model

Experiments were conducted on age- and gender-matched *Ctns*^*−/−*^ and *Ctns*^*+/+*^ littermates (C57BL/6 background^8^^,^^9^). Mice were maintained under temperature-and humidity-controlled conditions with 12 h light/12 h dark cycles with free access to appropriate standard diet in accordance with the institutional guidelines of National Institutes of Health Guide for the Care and Use of Laboratory Animals.

Mice aged 16 weeks were treated by daily intraperitoneal injection of Mito-TEMPO (1 mg kg^−1^ body weight in NaCl 0.9%) or vehicle (NaCl 0.9%) (*n* = 4 for each group), and killed after 30 days. Kidney tissues and/or urines were collected for analyses. The mouse study protocols (University of Zurich) were approved by the local legal authority (Veterinary Office, Canton of Zurich, Switzerland).

### Renal function

The mice were placed overnight in metabolic cages with ad libitum access to food and drinking water; urine was collected over ice^9,22^. The urine parameters were measured using a UniCel DxC 800 pro Synchron (Beckman Coulter, Fullerton, CA, USA), whereas Clara cell protein (CC16) concentration was measured in duplicate by enzyme-linked immunosorbent assay (BIOMATIK EKU03200).

### Kidney isolation and primary cultures of mouse PT cells

The kidneys were collected from *Ctns*^*+/+*^ and *Ctns*^*−/−*^ mice: one kidney was split transversally and one half was fixed and processed for immunostaining, whereas the other half was flash frozen, homogenized by Dounce homogenizer in 1 ml of RIPA buffer that contains protease and phosphatase inhibitors, and processed for western blot analysis^9,22^. The contralateral kidney was taken to generate primary cultures of mPTCs^22^. Freshly micro-dissected PT segments were seeded onto collagen-coated chamber slides (C7182, Sigma-Aldrich) and/or collagen-coated 6- or 24-well plates (145380 or 142475, Thermo Fisher Scientific), and cultured at 37 °C and 5% CO_2_ in Dulbecco’s modified Eagle’s medium/F12 (21041-025, Thermo Fisher Scientific) with 0.5% dialyzed fetal bovine serum (FBS), 15 mM HEPES (H0887, Sigma-Aldrich), 0.55 mM sodium pyruvate (P2256, Sigma-Aldrich), 0.1 ml l^−1^ non-essential amino acids (M7145, Sigma-Aldrich), hydrocortisone, human epidermal growth factor, epinephrine, insulin, triiodothyronine, transferrin (TF), and gentamicin/amphotericin (Single Quots kit, CC-4127, Lonza), pH 7.40, 325 mOsm kg^−1^. The medium was replaced every 48 h. Confluent monolayers of mPTCs were expanded from the tubular fragments after 6–7 days, characterized by a high endocytic uptake capacity. These cells were mycoplasma free. All experiments were performed on confluent monolayers grown on chamber slides or plates.

### Starvation of primary PT cells and treatments

Serum and amino acid removal was performed by washing mPTCs with Hank’s balanced salt solution (55021 C, Sigma-Aldrich) and placing them in nutrient-deprived medium. Where indicated, autophagy was inhibited by addition of either PIK3C3/Vps34 inhibitor SAR-405 (5 μM in cell culture medium for 16 h, unless otherwise specified^59^) or Spautin-1 (25 μM in cell culture medium for 16 h^60^). Lysosomal proteolysis was inhibited by addition of BfnA1 (250 nM for 4 h, unless otherwise stated). Where indicated, oxidative stress was triggered by treating mPTCs with either hydrogen peroxide (0.5 mM for 1 h) or with ROT (250 nM for 8 h). Where indicated, mPTCs were treated with either the mitochondria-targeted oxidant scavenger Mito-TEMPO (10 μM in culture medium for 12 h, Enzo Life Sciences) or with a selective Src-family kinase inhibitor SU6656 (5 μM in culture medium for 12 h, Sigma Aldrich). Afterwards, the cells were processed and analyzed as described below.

### Adenovirus transduction in primary PT cells

For RNA interference studies, the adenovirus constructs include scrambled short hairpin or shRNAs encoding individually mouse *Atg7* or encoding mouse *Ybx3/Csda*, or encoding mouse *Gna12* or encoding mouse *Tjp1*. For expression studies, adenovirus constructs include cytomegalovirus (Null) or particles individually carrying mouse GFP-tagged-*Map1lc3b* or carrying mouse red fluorescent protein (RFP)/2 × FLAG tagged*-Lamp1* or carrying GFP tagged-*Csda* or carrying human HA-tagged-*TJP1* or carrying mouse HA-tagged-*Ctns*. The aforementioned adenovirus constructs were purchased from Vector Biolabs (University City Science Center, Philadelphia, USA).

The primary mouse PT cells were plated onto collagen-coated chamber slides or 24-well or 6-well tissue culture plates. Adenovirus transduction was performed 24 h after plating when the cells reached ~ 70–80% confluence. The cells were subsequently incubated for 16 h at 37 °C with culture medium containing the virus at the concentration (0.2125 × 10^9^ PFU ml^−1^). The cells were afterwards challenged with fresh medium every 2 days, cultured for 5 days (unless otherwise specified), and collected for analyses.

### Quantitative real-time PCR

Total RNA was extracted from mouse and zebrafish kidney tissues using Aurum Total RNA Fatty and Fibrous Tissue Kit (Bio-Rad, Hercules, CA). DNAse I treatment was performed to eliminate genomic DNA contamination. Total RNA was extracted from primary cell cultures with RNAqueous kit (Applied Biosystems, Life Technologies). One microgram of RNA was used to perform the reverse transcriptase reaction with iScript cDNA Synthesis Kit (Bio-Rad). Changes in mRNA levels of the target genes were determined by relative reverse transcriptase-quantitative PCR with a CFX96 Real-Time PCR Detection System (Bio-Rad) using iQ SYBR Green Supermix (Bio-Rad). The analyses were performed in duplicate with 100 nM of both sense and anti-sense primers in a final volume of 20 µl using iQ SYBR Green Supermix (Bio-Rad). Specific primers were designed using Primer3 (Supplementary Table 1 and 2). PCR conditions were 95 °C for 3 min followed by 40 cycles of 15 s at 95 °C, 30 s at 60 °C. The PCR products were sequenced with the BigDye terminator kit (Perkin Elmer Applied Biosystems) using ABI3100 capillary sequencer (Perkin Elmer Applied Biosystems). The efficiency of each set of primers was determined by dilution curves (Supplementary Table 1 and 2). The program geNorm version 3.4 was applied to characterize the expression stability of the candidate reference genes in kidneys and six reference genes were selected to calculate the normalization factor^61^. The relative changes in targeted genes over *Gapdh* mRNAs were calculated using the 2^−ΔΔ^Ct formula.

### Endocytosis assay

The endocytic uptake was monitored in mPTCs cells following incubation for 60 min at 4 °C with 50 μg ml^−1^ bovine serum albumin (BSA)–Alexa-Fluor-647 (A34785, Thermo Fisher Scientific) or with 50 μg ml^−1^ BSA–Alexa-Fluor-488 (A13100, Thermo Fisher Scientific) in complete HEPES-buffered Dulbecco’s modified Eagle’s medium. The cells were given an acid wash and warmed to 37 °C in growth cell medium for 15 min before being fixed and processed for immunofluorescence analyses^22^.

### Lysosomal activity

The detection of lysosomal activity was performed in live mPTCs using Bodipy-FL-PepA (P12271, Thermo Fischer Scientific) or Magic Red-(RR)_2_ substrate (MR-CtsB; 938, Immunochemistry Technologies) according to the manufacturer’s specifications. The cells were pulsed with 1 μM Bodipy-FL-Pepstatin A or with 1 μM Magic Red-(RR)_2_ in Live Cell Imaging (A14291DJ, Thermo Fischer Scientific) medium for 1 h at 37 °C followed by fixation and immunostaining with anti-LAMP1 or anti-HA antibody, and subsequently analyzed by confocal microscopy^22^.

The lysosome-based processing was measured in zebrafish embryos using bovine β-lactoglobulin (Sigma-Aldrich). β-Lactoglobulin was tagged with Cy5 using TM2 Ab labeling kit (Amersham) in accordance with the manufacturer’s instructions. After anesthesia (0.2 mg ml^−1^ tricaine (3-amino benzoic acid ethyl ester, Sigma-Aldrich), *cdh17*::GFP-expressing *ctns* zebrafish embryos were injected at 5 dpf with Cy5-β-lactoglobulin (5 μg μl^−1^) via the common cardinal vein. Tracer injection into larvae blood was validated by monitoring the fluorescent vessels after injection. Larvae were incubated in fresh E3 medium for 20 or 120 min and subsequently fixed overnight in 2% paraformaldehyde (PFA)/phosphate-buffered saline (PBS) containing 0.1% Tween-20, and analyzed by fluorescent microscope (Zeiss, Germany).The fluorescence intensities and the numbers of β-lactoglobulin^+^ structures were quantified by using cell image analysis software Imaris (Bitplane).

### Extracellular flux analysis and metabolic measurement

OCR was measured in XFp Extracellular Flux Analyzers (Agilent Seahorse Biosciences) in mPTCs incubated with XF-Base Medium (non-buffered RPMI 1640 containing either 2 mM l-glutamine, 1 mM sodium pyruvate and 10 mM glucose, pH 7.4). Three measurements were assessed under basal conditions and upon addition of 2 μM Oligomycin (Oligo), 0.5 μM FCCP, and 1 μM ROT/Antimycin-A (ANT). All the reagents were provided by XFp Cell Mito Stress Test Kit (Agilent Seahorse Biosciences). OCR measurements were normalized to the numbers of the cells (TC10 automated cell counter, Bio-Rad).

### Mitochondrial membrane potential measurement

The mitochondrial membrane potential (Δψ) was measured in according with the manufacturer’s specifications. The cells were pulsed with 50 nM TMRM perchlorate (T668 Thermo Fisher Scientific) for 30 min in live-cell imaging at 37 °C. After washing, the cells were subsequently analysed by confocal microscopy^62^. The fluorescence intensity was quantified by using the open-source cell image analysis software CellProfiler^63^ as described below.

### ROS detection

The cells were pulsed with 5 µM CellROX Green Reagent (C10444, Thermo Fisher Scientific) or with 2.5 μM MitoSOX Red Mitochondrial Superoxide Indicator (M36008, Thermo Fisher Scientific) for 10 min in live-cell imaging at 37 °C. After washing, the cells were subsequently analysed by confocal microscopy^62^. The fluorescence intensity was quantified by using the open-source cell image analysis software CellProfiler^63^ as described below.

### Immunofluorescence and confocal microscopy

Fresh mouse kidneys were fixed by perfusion with 50–60 ml of 4% PFA in PBS (158127, Sigma-Aldrich), dehydrated and embedded in paraffin at 58 C°. Paraffin blocks were sectioned into consecutive 5 μm-thick slices with a Leica RM2255 rotary microtome (Thermo Fisher Scientific) on Superfrost Plus glass slides (Thermo Fisher Scientific). Before staining, slides were deparaffinized in changes of CitriSolv (22-143-975, Thermo Fisher Scientific) and 70% isopropanol. Antigen retrieval was accomplished by incubating in sodium citrate buffer (1.8% 0.1 M citric acid, 8.2% 0.1 M sodium citrate, in distillated water, pH 6.0) in a rice cooker for 30 min. The slides were quenched with 50 mM NH_4_Cl, blocked with 0.5% BSA in PBS Ca/Mg (D1283, Sigma-Aldrich) for 30 min and stained with primary antibodies specific for PCNA, LRP2, LC3, and SQSTM1 diluted in blocking buffer overnight at 4 °C. After two washes in 0.1% Tween 20 (v/v in PBS), the slides were incubated with the corresponding fluorophore-conjugated Alexa secondary antibodies (Invitrogen) diluted in blocking buffer at room temperature for 1 h and counterstained with 1 μg Biotinylated Lotus Tetragonolobus Lectin (LTL; B-1325 Vector Laboratories) and 1 µM 4’,6-diamino-2-phenylindole dihydrochloride (DAPI; D1306, Thermo Fischer Scientific). The slides were mounted in Prolong Gold Anti-fade reagent (P36930, Thermo Fisher Scientific), acquired on Leica SP8 confocal laser scanning microscope (Center for Microscopy and Image Analysis, University of Zurich) equipped with a Leica APO × 63 NA 1.4 oil-immersion objective at a definition of 1,024 × 1,024 pixels (average of 8 or 16 scans), adjusting the pinhole diameter to 1 Airy unit for each emission channel to have all of the intensity values between 1 and 254 (linear range). The micrographs were processed with Adobe Photoshop (version CS5, Adobe System, Inc., San Jose, USA) software. Quantitative image analysis was performed by selecting randomly ∼ 5 visual fields per each slide that included at least three to five PTs (LTL positive), using the same setting parameters (i.e., pinhole, laser power, and offset gain and detector amplification below pixel saturation). The numbers of LC3or SQSTM1-positive structures per PT (marked by LTL) were counted by using the open-source cell image analysis software CellProfiler^63^.

The mPTCs were fixed for 10 min with 4% PFA in PBS, quenched with 50 mM NH_4_Cl, and permeabilized for 20 min in blocking buffer solution containing 0.1% Triton X-100 and 0.5% BSA dissolved in PBS. Subsequently, mPTCs were incubated overnight with the appropriate primary antibodies at 4 °C. After repeated washing with PBS, the slides were incubated for 45 min with the suitable fluorophore-conjugated Alexa secondary antibodies (Invitrogen), counterstained with 1 µM DAPI for 5 min, mounted with the Prolong Gold Anti-fade reagent, and analyzed by a Leica SP8 confocal laser scanning microscope (Center for Microscopy and Image Analysis, University of Zurich) using the settings described above. Quantitative image analysis was performed by selecting randomly five visual fields pooled from biological triplicates, with each field including at least 20–25 cells, using the same setting parameters (i.e., pinhole, laser power, and offset gain and detector amplification below pixel saturation). The 3D reconstructions of LAMP1-positive vesicles were generated in Imaris software using full confocal *z*-stacks (around 20) of each cell. The quantitative cell image analyses were performed using the open-source cell image analysis software CellProfiler^63^. In particular, the specific module “Measure-Object-Intensity-Distribution” was used to score the number and the vesicle size of LAMP1 or CtsD-positive structures and the fractions of LAMP1-positive structures contained into perinuclear region (area within 10 μm of the nucleus) and peripheral region (area ≥ 15 μm from the nucleus), respectively. The pipeline “Speckle counting” was used to identify smaller objects (LC3 or SQSTM1, or Prohibitin or BSA, or CtsD or MR-Ctsb, or ATG16L-positive structures) surrounding larger objects and to perform per-object aggregate measurements. The pipeline “Cell/particle counting and scoring the percentage of stained objects” was used to score either the numbers of LAMP1^+^ vesicles containing LC3^+^ autophagosomes or PepA, or CtsD ^+^ vesicles or tight junction-ZO-1^+^ structures, or Prohibitin^+^ mitochondria, or the numbers of CtsD^+^ vesicles containing MR-CtsB peptides or Golgi protein GM130. The “Cytoplasm–Nucleus Translocation Assay” was used to score the numbers of ZONAB or BrdU or PCNA-positive nuclei.

### EM and tomography

Zebrafish larvae and cultured mPTCs were fixed in 2% PFA/2.5% glutaraldehyde (for zebrafish) and 4% PFA/0.1% glutaraldehyde (for cells) in 100 nM sodium cacodylate, at pH 7.43, dehydrated, and embedded in LR-White resin (LADD Research Industries). The grids were viewed on a Philips CM100 transmission electron microscope at 80 kV. Autophagic vacuoles were identified and categorized as autophagosomes or autophagolysosomes according to standard criteria^23^. The term autophagic vacuole was used when the differentiation between autophagosome and autophagolysosomes was not possible. The number of autophagic vacuoles was determined by using i-TEM software (Olympus, Germany). For electron tomography, 200–250 nm-thick sections were collected on formvar-coated copper slot grids using a Leica EM UC7 ultramicrotome (Leica Microsystem GmbH, Vienna, Austria). Colloidal gold particles (10 nm) were applied as fiducials on both surfaces of the grids and the samples were imaged on a 200 kV Tecnai G2 20 electron microscope (FEI, Eindhoven) at a magnification of × 11,500, resulting in pixel sizes of 1.95 nm. Single, tilted image series ( ± 60° according to a Saxton scheme with the initial tilt step of 2°) were acquired using Xplorer3D (FEI) with an Eagle 2,048 × 2,048 CCD camera (FEI). Tilted series alignment, tomographic reconstructions, image segmentation, and visualization were done with the IMOD software package.

### Correlative light EM

The cells were grown on finder grids and prepared for confocal microscopy analyses. Z-stacks of cells of interest were taken with the PerkinElmer UltraView ERS confocal microscope. The coordinates of the cells on the finder grid were determined by bright-field microscopy. Cells were fixed in 1% glutaraldehyde in 0.1 M cacodylate buffer (Sigma) and post-fixed with 1.5% potassium ferricyanide, 1% OsO_4_ in 0.1 M cacodylate buffer. Cells were stained overnight with 0.5% uranyl acetate, dehydrated in ethanol, and embedded in epon. After baking for 48 h at 60 °C, the resin was released from the glass coverslip by temperature shock in liquid nitrogen. Serial sections (70–90 nm) were collected on carbon-coated formvar slot grids and imaged with a Zeiss LEO 512 electron microscope. Images were acquired by a 2k × 2k bottom-mounted slow-scan Proscan camera controlled by EsivisionPro 3.2 software.

### Soluble and insoluble fractionation

The cells were lysed in buffer containing 50 mM Tris-HCl, pH 7.5, 150 mM NaCl, 0.1% SDS, 1% Triton X-100, 1% sodiumdeoxycholate supplemented with protease and phosphatase inhibitors, and centrifuged at 16,000 r.p.m. at 4 °C for 20 min, to collect the soluble fraction (supernatant). The pellet was suspended in a buffer containing 4% SDS and 20 mM HEPES, pH 7.5, protease and phosphatase inhibitors, and further centrifuged at 18,000 r.p.m. at room temperature for 10 min, to collect the insoluble fraction (supernatant). The samples were boiled at 95 °C for 5 min and analyzed by western blotting.

### Immunoprecipitation

The cells were lysed in a buffer containing 25 mM Tris/HCl adjusted to pH 7.4, 150 mM NaCl, 1% NP-40, and 1 mM EDTA, 5% glycerol, protease and phosphatase inhibitors for 10 min at 4 °C. Lysates were centrifuged at 12,000 r.p.m. for 10 min and the supernatants were incubated with 1.5 μg of primary anti-ZO-1 antibody for 16 h at 4 °C. After addition of 50 μl of protein A sepharose beads (P9424, Sigma), lysates were incubated at 4 °C for 12 h with gentle shaking. Agarose beads were washed for three times and then collected through centrifugation at 4,000 r.p.m. for 1 min. Beads were reduced by the addition of 35 μl of Laemmli sample buffer, heated at 95 °C for 5 min, and resolved on 10% SDS-polyacrylamide gel electrophoresis (PAGE) gel, and analyzed by western blotting.

### Western blotting

Proteins were extracted from mouse or zebrafish kidney tissues or primary cultured cells, lysed using a buffer, which contains protease (1836153001, Roche) and phosphatase inhibitors (04906845001, PhosSTOP Sigma), followed by sonication and centrifugation at 12,000 r.p.m. for 10 min at 4 °C. The samples were thawed on ice, normalized for protein (20 μg per lane), dissolved in Laemmli sample buffer, and separated by SDS-PAGE under reducing conditions. After blotting onto polyvinylidene difluoride and blocking with 5% non-fat milk (1706404, Bio-Rad Laboratories) diluted in PBS, the membranes were incubated overnight at 4 °C with primary antibody, washed, incubated with peroxidase-labeled secondary antibody, and visualized with enhanced chemiluminescence (WBKLS0050, Millipore, Life technologies). For re-probing, the membranes were rinsed, incubated for 30 min at 55 °C in a stripping buffer (62.5 mmol l^−1^ Tris-HCl, 2% SDS, 100 mM mercaptoethanol, adjusted to pH 7.4), before incubation with primary antibodies. Quantitative analyses were performed by scanning the blots and measuring the relative density of each band normalized to β-actin, GAPDH, or α-tubulin with ImageJ software. Unprocessed scans of original blots are shown in Supplementary Fig. 13.

### Proximity ligation assay

The PLA assay was performed using the Duolink in situ reagents (Olink Biosciences) in according with the manufacturer’s specifications. The cells were fixed with 4% PFA in PBS for 15 min at room temperature. After blocking with 0.1% Triton X-100 and 0.5% BSA dissolved in PBS, the cells were incubated overnight at 4 °C with primary antibodies. Following incubation with Duolink PLA anti-rabbit Plus and anti-mouse Minus probes (1:5 dilution, Olink Bioscience) at 37 °C for 1 h, ligation, rolling circle amplification, and detection were performed using the Duolink In Situ Detection Reagents Red (Olink Bioscience). Nuclei were counterstained with DAPI. PLA signals were documented by a Leica SP8 confocal microscope and quantified by using cell image analysis software CellProfiler^63^.

### PtdIns-3P detection

The cells were fixed with 2% formaldehyde in PBS for 15 min at room temperature. After washing with PBS containing 50 mM NH_4_Cl, the cells were permeabilized for 5 min by the addition of 20 μM digitonin in bufferA (20 mM Pipes pH 6.8, 137 mM NaCl, 2.7 mM KCl). Digitonin was removed by three rinses in bufferA and cells were blocked for 45 min with buffer A supplemented with 5% (v/v) FBS and 50 mM NH_4_Cl. Monoclonal mouse anti-PtdIns-3-P antibody^64^ was diluted in buffer with 5% FBS for 1 h (1:300). After washing with buffer A for three times, anti-mouse IgG secondary antibodies were applied in buffer with 5% FBS (1:400) for 30 min. Cells were underwent to post fixation for 5 min in 2% PFA, washed with PBS containing 50 mM NH_4_Cl, and then mounted with the Prolong Gold Anti-fade reagent. PtdIns-3-P levels were analyzed by using a Leica SP8 confocal laser scanning microscope and quantified by using CellProfiler^63^.

### Proliferation assay

To measure cell proliferation, the cells were seeded in 24-well plates at a density of 2.0 × 10^4^ cells per well. The cells were cultured for 3 days and cell medium was renewed daily. Afterwards, the cells were trypsinized every 24 h and quantified using the Countess automated cell counter TC10 automated cell counter (BIO-RAD). The time-course experiments were repeated three times using cells derived from three individual mice per each group. Where indicated, cells were incubated with BrdU (1.5 μg ml^−1^; Sigma-Aldrich) in accordance with the manufacturer’s protocol. BrdU-labeled cells were detected by immunostaining using rat anti-BrdU antibody (Oxford Biotechnology; 1:500), followed by the specific secondary biotinylated goat anti-rat antibody (1:300; Jackson Immunoresearch) and mounted with the Prolong Gold Anti-fade reagent. The slides were analyzed by using a Leica SP8 confocal laser scanning microscope and quantified by using CellProfiler.

### Cystine measurement

Kidney tissues from mice kidney tissue or from zebrafish embryos, or primary cultured cells were homogenized and lysed with N-ethylmaleimide (NEM) solution containing 5.2 mmol l^−1^ *N*-ethylmaleide in 10 mmol l^−1^ potassium phosphate buffer adjusted to pH 7.4. The lysates were collected and precipitated with sulfosalicylic acid (12% w/v) and centrifuged at 10,000 r.p.m. for 10 min at 10 °C. The resulting supernatant was dissolved in citrate loading buffer (Biochrom Ltd, Cambridge, UK) and 50 µl of this solution was analyzed by Biochrom 30 Plus Amino Acid Analyzer (Biochrom Ltd). The protein pellet was dissolved in 0.1 mol l^−1^ NaOH solution and the protein concentration was determined by Biuret method. The concentration of amino acids was measured by using a lithium high performance physiological column (Biochrom) followed by post-column derivatization with ninhydrin. The amino acids were identified according to the retention time and the ratio of the area between the two wavelengths (570 nm and 440 nm) and quantified by using EZChrom Elite software (Agilent Technologies Inc., Pleasanton, California, USA). Cystine concentration was normalized to the protein concentration and reported in nmol per mg protein. N-ethylmaleimide

### Data analysis and statistics

The quantitative data were expressed as means ± SEM. Differences between experimental groups were evaluated using one-way analysis of variance followed by Bonferroni or Dunnet’s post hoc test, when appropriate. When only two groups were compared, unpaired or paired two-tailed Student’s *t*-tests were used as appropriate. No statistical methods were used to predetermine the sample size. We estimated the sample size considering the variation and mean of the samples. Assumptions for statistical analyses were met (that is, normal distribution and equal variance). The sample size (number of cells or number of biological replicates derived from distinct mice or zebrafish) of each experimental group is described in figure legends. The results are representative of at least three independent experiments, unless specified in the figure legends. None of the samples/animals was excluded from the experiment, and the animals were not randomized. The investigators were not blinded to allocation during the experiments and outcome assessment. GraphPad Prism software was used for all statistical analyses. Statistical significance was set at a *P* < 0.05.

### Data availability statement

The data that support the findings of this study are available on reasonable request from the corresponding authors (O.D. and A.L.)

## Electronic supplementary material

Supplementary Information

Description of Additional Supplementary Files

Supplementary Movie 1

Supplementary Movie 2

Supplementary Movie 3
