# Supplementary material for: Impaired autophagy bridges lysosomal storage disease and epithelial dysfunction in the kidney
Source: Nat Commun. 2018 Jan 11;9:161. doi: 10.1038/s41467-017-02536-7 (PMC5765140; doi:10.1038/s41467-017-02536-7)
Supplement: Supplementary file 1 — Supplementary Information [file 41467_2017_2536_MOESM1_ESM.pdf]

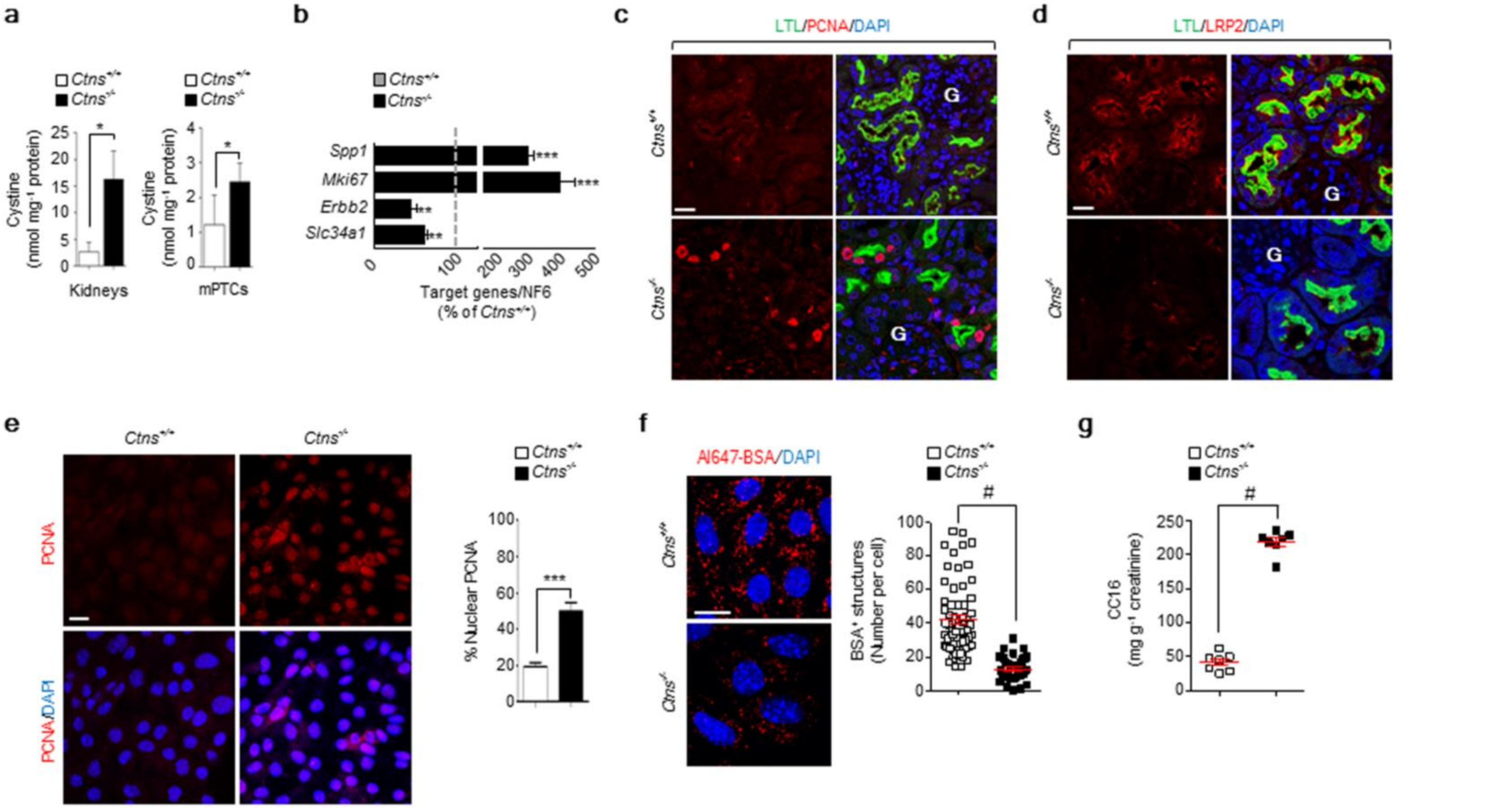

Supplementary Figure 1. Cystine storage, proliferation and dedifferentiation in *Ctns*$^{-/-}$ kidneys and their derived PT cells. (**a**) Cystine levels in kidney cortex from 24-week-old *Ctns* mice (left panel) and in their derived primary mPTCs (right panel) were assessed by HPLC (n=5 per group). (**b**) The mRNA levels of *Slc34a1*, *Erbb2*, *Mki67*and *Spp1* in *Ctns* kidneys were analysed by real-time PCR (n=4 kidneys per group). (**c**) *Ctns* kidneys were immunostained with anti-PCNA (red; left panel) or (**d**) with anti-LRP2 (red; right panel) antibodies and stained afterwards with Lotus Tetragonolobus Lectin (LTL; proximal tubule marker, green). Scale bar, 50 µm. (**e**) *Ctns* mPTCs were immunostained with anti-PCNA antibody (red) and analysed by confocal microscopy. Quantification of numbers of PCNA^{+} nuclei (expressed as percentage of total nuclei) obtained from 5 randomly selected fields per condition, with each containing ~20-25 cells. (**f**) *Ctns* mPTCs were loaded with Al647-BSA (50µg ml^{-1} for 15 min at 37°C) and analysed by confocal microscopy. Quantification of numbers of Al647-BSA^{+} structures (n=45-65 randomly selected cells pooled from three mouse kidneys per condition; each point representing the number of BSA^{+} structures in a cell). (**g**) CC16 protein levels in urine from 24-week-old *Ctns* mice were measured by ELISA (n=7 mice per group). Plotted data represent mean ± SEM; two tailed unpaired Student's *t* test, **P*<0.05; ***P*<0.01; ****P*<0.001; $^{\#}$*P*<0.0001 relative to *Ctns*$^{+/+}$ kidneys or mPTCs. Nuclei counterstained with DAPI (blue). Unless otherwise stated, scale bars are 10µm.

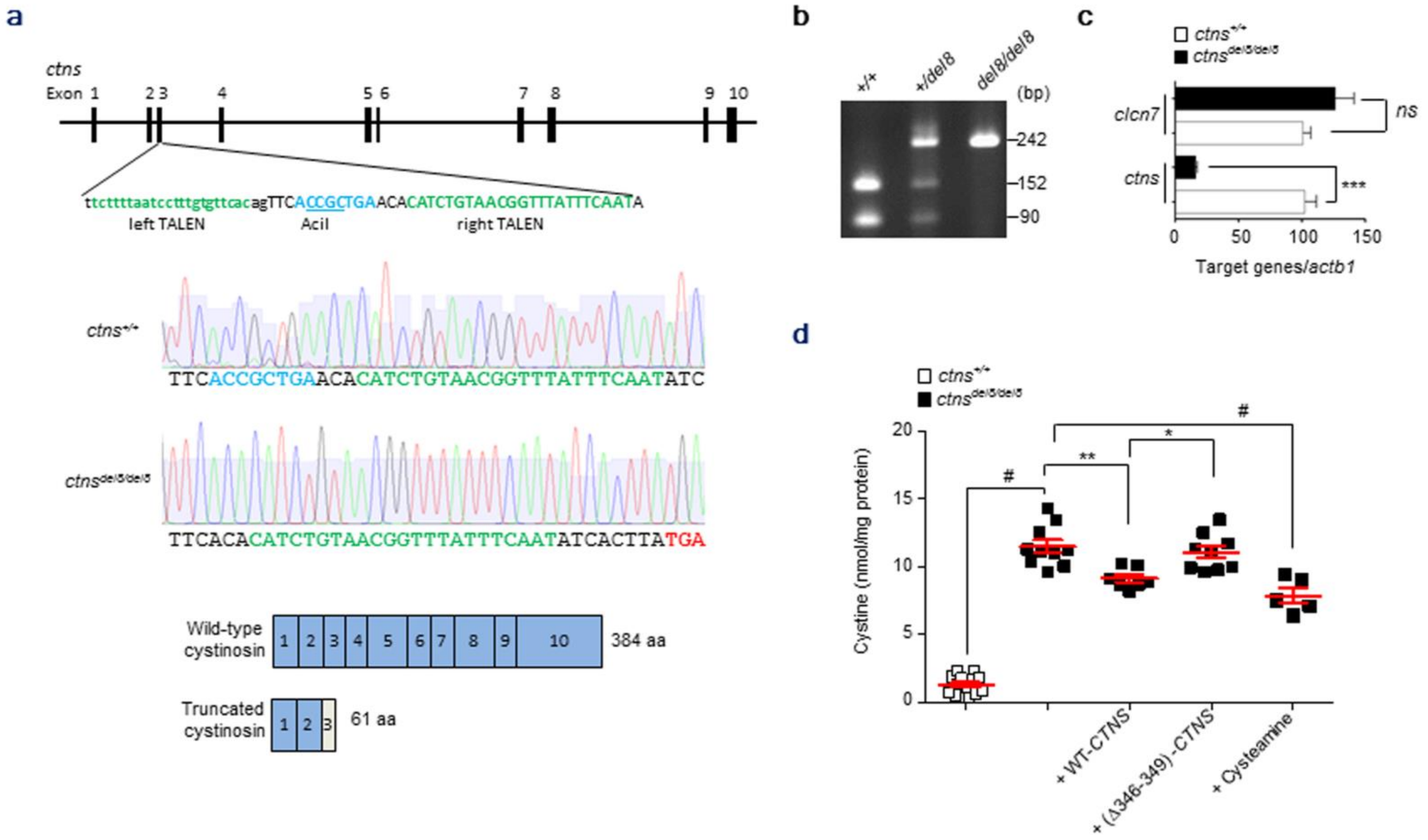

Supplementary Figure 2. Generation and validation of *ctns* knock-out zebrafish model. (**a**) TALEN-induced deletion (blue) generates a frameshift of the open reading frame, resulting in premature stop codon (TGA) within the exon 3 of *ctns* gene (middle panel) and producing a truncated protein of 61 amino acids. The underlined sequence represents the AciI site which is used for the detection of the TALEN-induced deletion; the sequence in green indicates both the left and right TALEN targeting site. (**b**) AciI digestion of PCR products after the amplification of the TALEN targeting region using genomic DNA extracted from the caudal region of wild-type (+/+), heterozygous (+/*del8*) and homozygous (*del8/del8*) zebrafish. Wild type allele: two lower bands 152bp + 90bp correspond to PCR products cut by AciI, mutant allele: upper band 242bp, resistant to AciI digestion. (**c**) The mRNA levels of *ctns* and *clcn7* were analysed by real-time PCR in zebrafish kidneys; n=7 *ctns*$^{+/+}$, n=8 *ctns*$^{del8/del8}$; two tailed unpaired Student's *t* test, ***P<0.001 relative to *ctns*$^{+/+}$ zebrafish; *ns*, not significant. (**d**) Cystine levels were assessed by HPLC in *ctns*$^{+/+}$ and in *ctns*$^{del8/del8}$ zebrafish larvae injected with wild type or mutant (Δ346-349) human *CTNS* mRNA or incubated in the E3 medium in presence of 1mM cysteamine; n=14 *ctns*$^{+/+}$ zebrafish, n=10 *ctns*$^{del8/del8}$ zebrafish, n=6 *ctns*$^{del8/del8}$ zebrafish injected with wild-type *CTNS*, n=8 *ctns*$^{del8/del8}$ zebrafish injected with mutant *CTNS*, n=5 *ctns*$^{del8/del8}$ zebrafish treated with cysteamine. Plotted data represent mean ± SEM. One-way ANOVA followed by Bonferroni *post hoc* test. *P<0.05 relative to *ctns*$^{del8/del8}$ zebrafish injected with wild-type *CTNS*, **P<0.01 relative to *ctns*$^{del8/del8}$, $^{\#}P$<0.0001 relative to *ctns*$^{+/+}$ or untreated *ctns*$^{del8/del8}$.

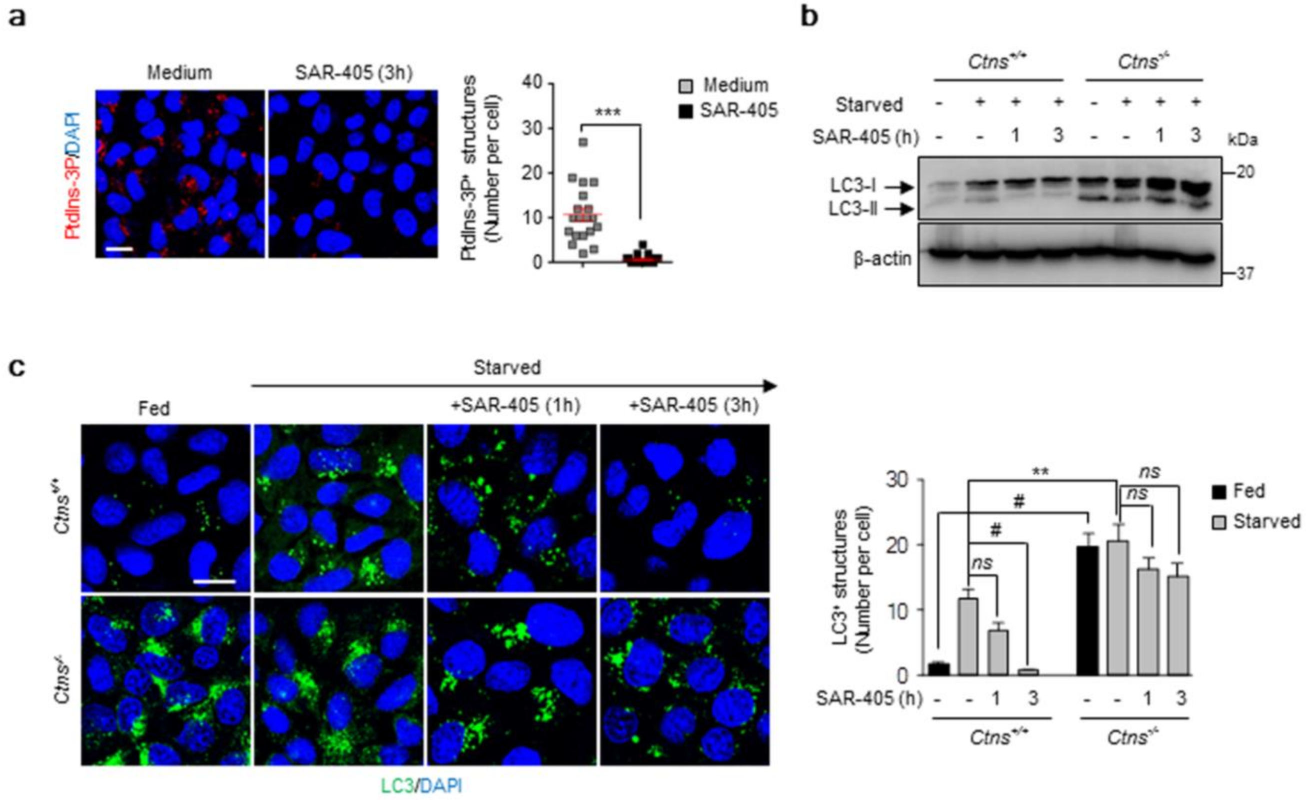

Supplementary Figure 3. Validation of the PIK3C3/Vps34 inhibitor SAR-405 and its effect on autophagy in *Ctns* PT cells. (**a**) mPTCs were exposed to SAR-405 (5μM, for 3h), immunostained with anti-PtdIns-3P antibody (red) and analysed by confocal microscopy. Quantification of numbers of PtdIns-3P$^+$ structures (n=20 cells pooled from three mouse kidneys per each condition; each point representing the number of PtdIns-3P$^+$ structures in a cell; mean±s.e.m; two tailed paired Student's *t*-test, ***P< 0.001 relative to untreated cells). (**b-c**) mPTCs were cultured in fed or in starved medium in presence of 5μM SAR-405 for the indicated times and subjected to western blotting and confocal microscopy analyses. (**b**) Representative western blotting of LC3 protein levels. β-actin was used as a loading control; n= 2 independent experiments. (**c**) The cells were immunostained with anti-LC3 antibody (green) and analysed by confocal microscopy. Quantification of numbers of LC3$^+$ structures (*n*=33 cells pooled from three *Ctns* kidneys per each condition; each point representing the number of LC3$^+$ structures in a cell; mean ± SEM; one-way ANOVA followed by Bonferroni's *post hoc* test, **P<0.01 relative to starved *Ctns*$^{+/+}$ mPTCs; $^{\#}P$<0.0001 relative to fed or starved *Ctns*$^{+/+}$ mPTCs; *ns*, not significant). Nuclei counterstained with DAPI (blue). Scale bars are 10 μm.

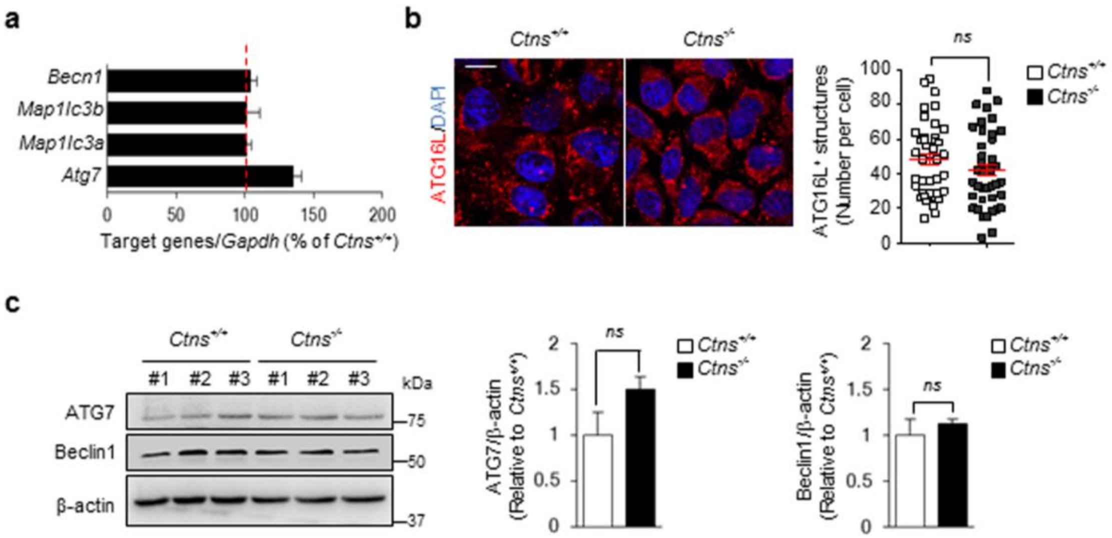

Supplementary Figure 4. Cystinosin deletion does not alter autophagosome biogenesis. (**a**) The mRNA levels of *Becn1, Map1lc3b, Map1lc3a* and *Atg7* in microdissected proximal tubules obtained from 24-week-old *Ctns* mouse kidneys were analysed by real-time PCR (n=3 *Ctns* kidneys per group). (**b**) Cells were immunostained with anti-ATG16L antibody (red) and analysed by confocal microscopy. Nuclei counterstained with DAPI (blue). Scale bar, 10μm. Quantification of numbers of ATG16L^{+} structures (n=40 cells pooled from three *Ctns* kidneys per each condition; each point representing the number of ATG16L^{+} structures in a cell; *ns*, not significant). (**c**) Western blotting and densitometric analyses of the protein levels of ATG7 and Beclin1 (n=3 independent experiments). β-actin was used as a loading control. Plotted data represent mean ± SEM; *ns*, not significant.

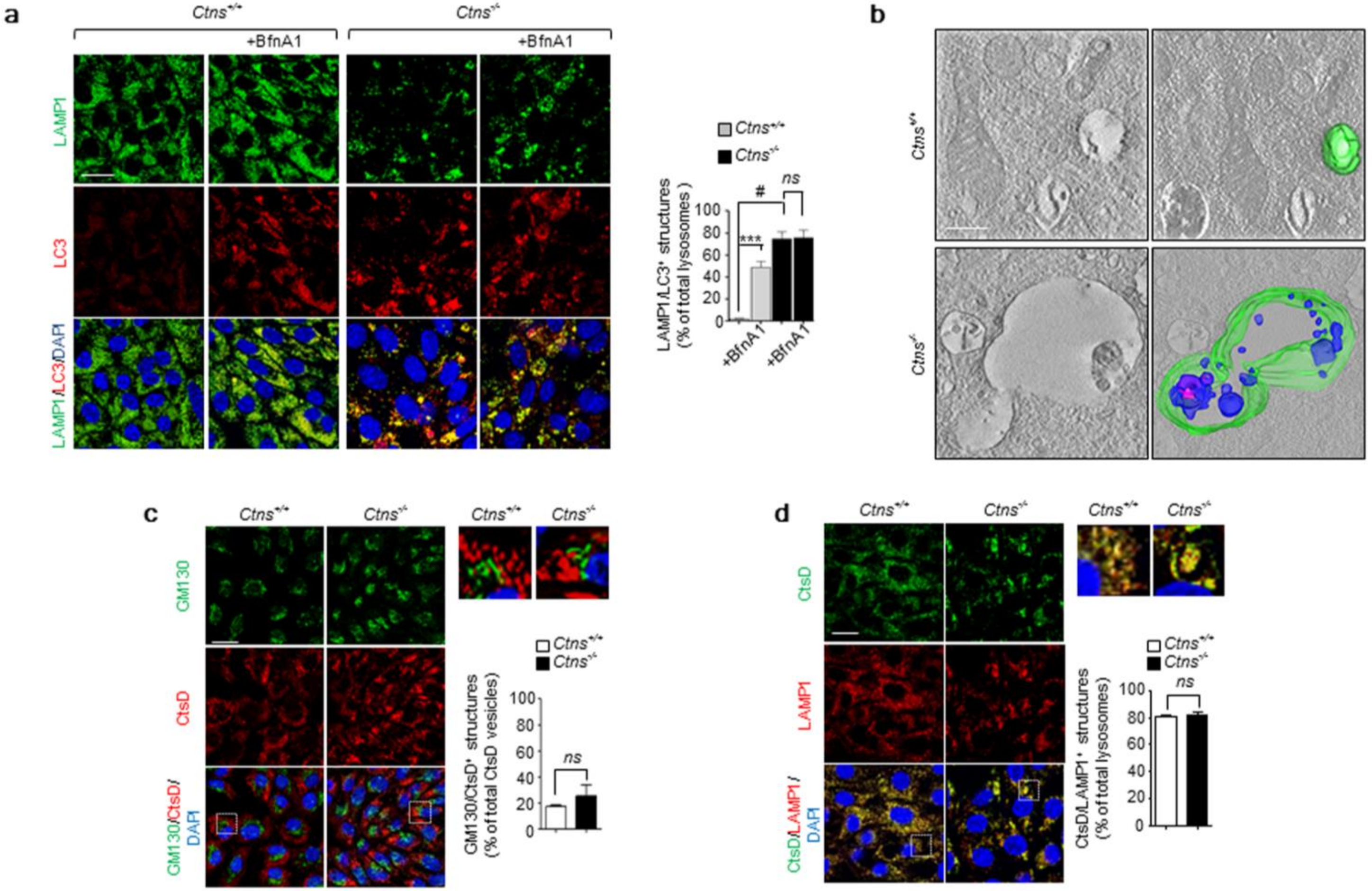

Supplementary Figure 5. Cystinosin deletion affects neither the autophagosome-lysosome fusion nor Golgi-to-lysosome trafficking of cathepsins. (**a**) Representative confocal micrographs and quantification of numbers of LC3/LAMP1$^+$ structures (expressed as percentage of total lysosomes) in cells cultured with non-saturating concentrations of BfnA1 (50 nM for 1h); n= 5 randomly selected fields per condition, with each containing ~20-25 cells; one way ANOVA followed by Bonferroni *post hoc* test, ***P<0.001 or $^{\#}P$<0.0001 relative to untreated *Ctns*$^{+/+}$ mPTCs; *ns*, not significant. (**b**) Representative electron tomography micrographs and 3-D reconstructed tomograms showing the presence of enlarged, single membranous structures (reminiscent of autolysosome; green) filled with undegraded cellular debris (blue and pink) in *Ctns*$^{-/-}$ mPTCs. (**c**) Cells were immunostained with anti-GM130 (Golgi marker; green) and anti-cathepsin-D (CtsD; red) antibodies or (**d**) with anti-CtsD (green) and anti-LAMP1 (late endosome/lysosome marker; red) antibodies. Yellow indicates colocalization. The dotted white squares show images at higher magnification. Quantification of numbers of GM130/CtsD$^+$ structures (expressed as percentage of total CtsD$^+$ structures) and of CtsD/LAMP1$^+$ structures (expressed as percentage of total LAMP1$^+$ structures); n=5 randomly selected fields per condition, with each containing ~20-25 cells. The plotted data show mean ± SEM; *ns*, not significant. Nuclei counterstained with DAPI (blue). Scale bars are 10μm in **a**, **c**, **d**, and 0.5 μm in **b**.

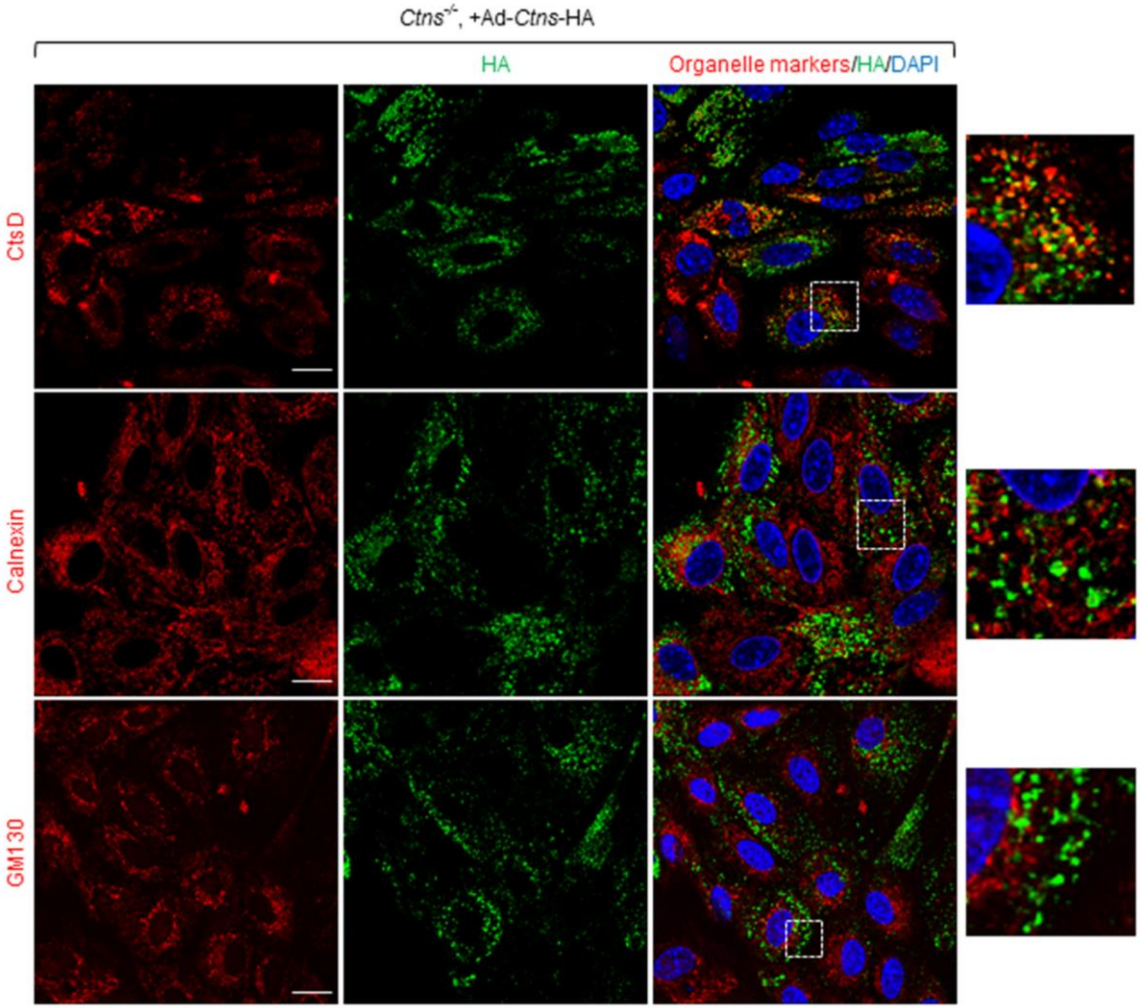

Supplementary Figure 6. Subcellular compartmentalization of rescued cystinosin in *Ctns*$^{-/-}$ cells. *Ctns*$^{-/-}$ mPTCs were transduced with hemagglutinin-tagged *Ctns* (HA-*Ctns*)-bearing adenoviral particles for 2 days. The cells were immunostained with anti-HA (green) and anti-CtsD (red; top panel) or with anti-HA and anti-Calnexin (red; middle panel) or with anti-HA and anti-GM130 (red; bottom panel) antibodies and analysed by confocal microscopy. Yellow indicates colocalization. The dotted white squares contain images at higher magnification. Nuclei counterstained with DAPI (blue). Scale bars are 10μm.

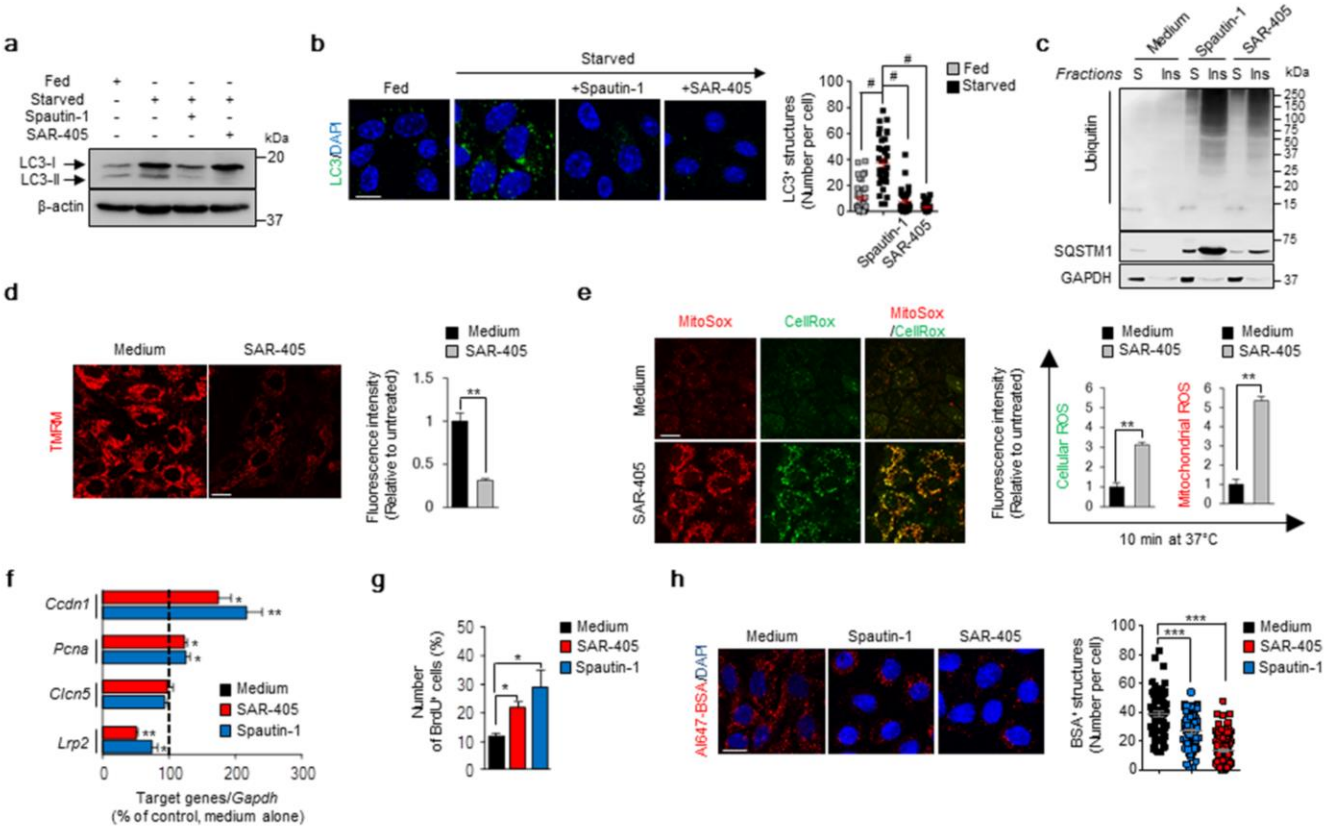

Supplementary Figure 7. Pharmacological blockage of autophagy causes mitochondria-derived oxidative stress, leading to proliferation and dedifferentiation of PT cells. (**a-b**) mPTCs were cultured in fed or starved medium (for 8h), or in starved medium in presence of PIK3C3/Vps34 inhibitor SAR-405 (5µM, for 8h) or autophagy inhibitor Spautin-1 (25µM, for 8h) or (**c-h**) in fed medium in presence or in absence of either SAR-405 (5µM, for 16h) or Spautin-1 (25µM, for 16h). (**a**) Representative western blotting of LC3 protein levels. β-actin was used as a loading control; n= 3 independent experiments. (**b**) Representative micrographs and quantification of numbers of $LC3^+$ structures (n=35 cells pooled from three mouse kidneys per condition; each point representing the number of $LC3^+$ structures in a cell). (**c**) Representative western blotting of soluble and insoluble fractions obtained from mPTCs and immunoblotted for ubiquitin, SQSTM1 and GAPDH; n= 2 independent experiments. (**d**) The cells were loaded with tetramethylrhodamine methyl ester (TMRM; mitochondrial membrane potential fluorescent probe, 50nM for 30min at 37°C) or (**e**) with both CellROX (cellular ROS indicator; 5µM for 10min at 37°C) and MitoSOX (mitochondrial ROS indicator; 2.5µM for 10min at 37°C) and analysed by confocal microscopy. Yellow indicates colocalization. (**f**) The mRNA levels of *Lrp2, Ccdn1, Clcn5* and *Pcna* were assessed by real-time PCR (n=4 independent experiments). (**g**) The cells were loaded with bromodeoxyuridine (BrdU, 1.5µg ml^{-1} for 16h at 37°C), immunostained with anti- BrdU antibody and analysed by confocal microscopy. Quantification of numbers of $BrdU^+$ cells (expressed as percentage of total nuclei). (**h**) Cells were loaded with Al647-BSA (50µg ml^{-1} for 15 min at 37°C) and analysed by confocal microscopy. Quantification of numbers of Al647-BSA^+ structures (n=68-105 randomly selected cells pooled from three mouse kidneys per condition; each point representing the number of BSA^+ structures in a cell). Plotted data represent mean ± SEM. The quantifications in **d**, **e** and **g** were obtained from 5 randomly selected fields per condition, with each containing ~20-25 cells. One-way ANOVA followed by Bonferroni's *post hoc* test, $^{\#}P<0.0001$ relative to mPTCs under fed or to starved conditions in **b.** Two tailed paired Student's *t* test, $^{**}P<0.01$ relative to untreated mPTCs in **d** and **e.** One-way ANOVA followed by Dunnett's *post hoc* test, $^{*}P<0.05$, $^{**}P<0.01$, $^{***}P<0.001$ relative to untreated mPTCs in **f, g** and **h**. Nuclei counterstained with DAPI (blue). Scale bars are 10µm.

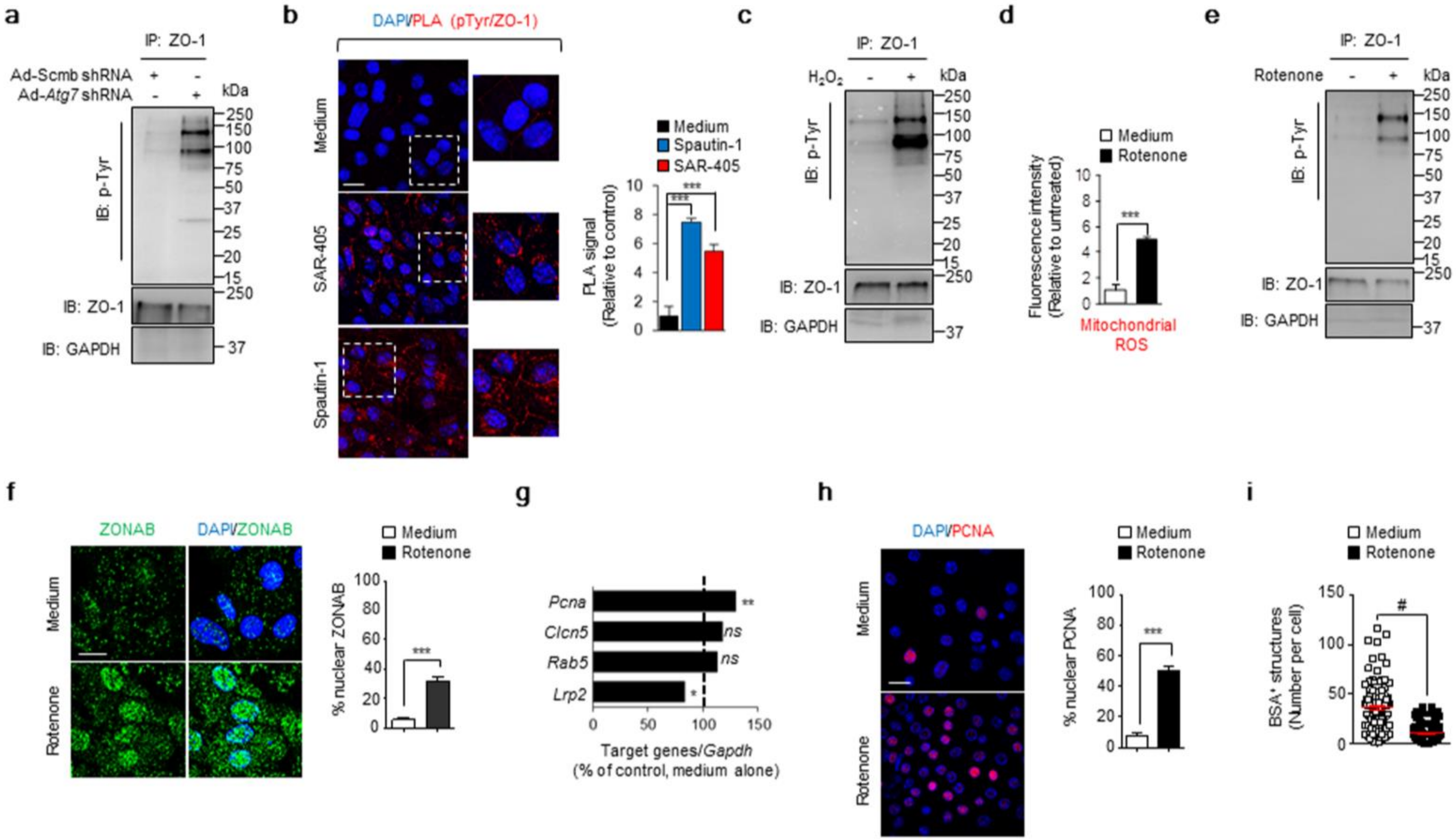

Supplementary Figure 8. Augmented phosphorylation of ZO-1 in PT cells experiencing autophagy failure or oxidative stress. mPTCs were transduced with Scrambled (Scmb) or *Atg7* adenoviral shRNAs for 5 days (**a**), or (**b**) cultured in presence of PIK3C3/Vps34 inhibitor SAR-405 (5µM, for 16h) or with the autophagy inhibitor Spautin-1 (25µM for 16h), or in presence (**c**) of H_2O_2 (hydrogen peroxide, 0.5mM for 1h) or (**d-e**) with mitochondrial complex I inhibitor Rotenone (250 nM for 8h). (**a,c,e**) Tight junction ZO-1 protein was immunoprecipitated (IP) from mPTCs and its phosphorylation rate was examined by western blotting, n=2 independent experiments. (**b**) Representative confocal micrographs and quantification of endogenous phosphorylation of ZO-1 by proximity ligation assay. One-way ANOVA followed by Dunnett's *post hoc* test, ***P<0.001 relative to untreated mPTCs. (**d**) The cells were loaded with MitoSOX (mitochondrial ROS probe; 2.5 µM for 10 min at 37°C) and analysed by confocal microscopy. (**f**) The cells were immunostained with anti-ZONAB (green) antibody and analysed by confocal microscopy. Quantification of the numbers of $ZONAB^+$ nuclei (expressed as percentage of total nuclei). (**g**) The mRNA levels of *Clcn5*, *Rab5*, *Lrp2* and *Pcna* were analysed by real-time PCR (n=4 independent experiments). (**h**) The cells were immunostained with anti-PCNA (red) antibody and analysed by confocal microscopy. Quantification of the numbers of $PCNA^+$ nuclei (expressed as percentage of the total nuclei). (**i**) The cells were loaded with Al647-BSA (50µg ml^{-1} for 15 min at 37°C) and analysed by confocal microscopy. Quantification of numbers of Al647-BSA^+ structures per cell (n=100 randomly selected cells pooled from three mouse kidneys per condition; each point representing the number of BSA^+ structures in a cell). Plotted data represent mean ± SEM. The quantifications in **d**, **f** and **h** were obtained from 5 randomly selected fields per condition, with each containing ~20-25 cells. Two tailed paired Student's *t* test, *P<0.05, **P<0.01, ***P<0.001, $^{\#}P$<0.0001 relative to untreated mPTCs in **d**, **f, g**, **h** and **i**; *ns*, not significant. Nuclei counterstained with DAPI (blue). Scale bars are 10µm.

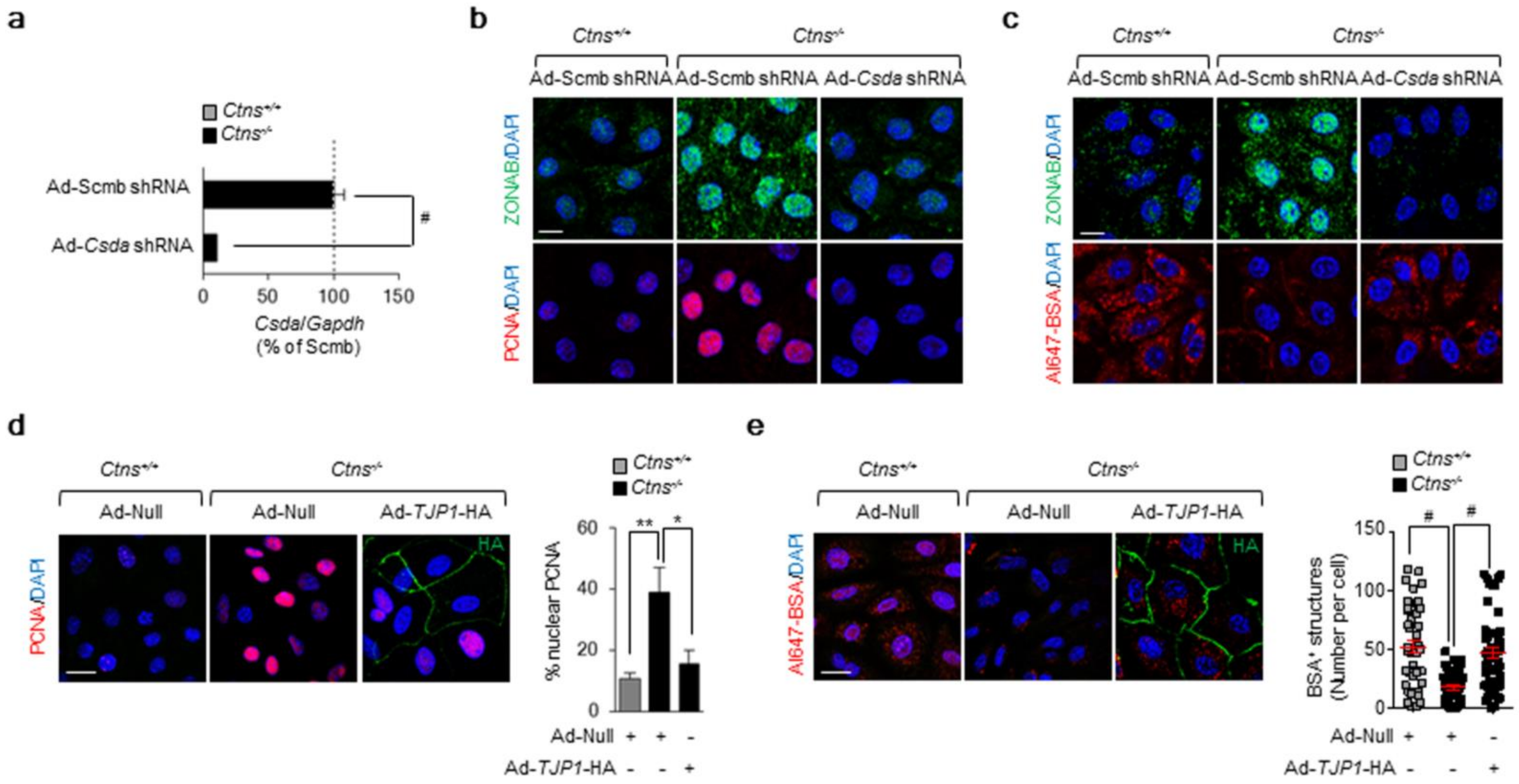

Supplementary Figure 9. Knockdown of *Csda* and the exogenous overexpression of *TJP1* in cystinosis PT cells rescue the epithelial function. (a-c) *Ctns* mPTCs were transduced with Scrambled (Scmb) or *Csda* adenoviral shRNAs for 5 days. (**a**) The mRNA levels of *Csda* were analysed by real-time PCR (n=4 independent experiments). (**b**) Representative micrographs of *Ctns* cells stained with anti-ZONAB (green; top panel) and anti-PCNA (red; bottom panel) antibodies or (**c**) loaded with Al647-BSA (red; bottom panel) and stained afterwards with anti-ZONAB (green; top panel) antibody. (**d-e**) *Ctns* cells were transduced with a Null or human HA-(*TJP1*)-bearing adenoviral particles for 2 days. (**d**) The cells were immunostained with anti-PCNA (red) and anti-HA (green) antibodies and analysed by confocal microscopy. Quantification of PCNA$^+$ nuclei (expressed as percentage of the total nuclei) obtained from 5 randomly selected fields per condition, with each containing ~20-25 cells. (**e**) The cells were loaded with Al647-BSA (red; 50µg ml^{-1}, for 15 min at 37°C), stained with anti-HA antibody (green) and analysed by confocal microscopy. Quantification of numbers of Al647-BSA$^+$ structures per cell (n=50 randomly selected cells pooled from three mouse kidneys per condition; each point representing the number of BSA$^+$ structures in a cell). Plotted data show mean ± SEM. Two tailed paired Student's *t* test, $^{\#}P$<0.0001 *Ctns*$^{-/-}$ transduced with Scmb shRNAs in **a**. One-way ANOVA followed by Bonferroni *post hoc* test, *P<0.05, **P<0.01 and $^{\#}P$<0.0001 relative to *Ctns*$^{-/-}$ transduced with Ad-Null or relative to *Ctns*$^{+/+}$ transduced with Ad-Null. Nuclei are counterstained with DAPI (blue). Scale bars are 10µm.

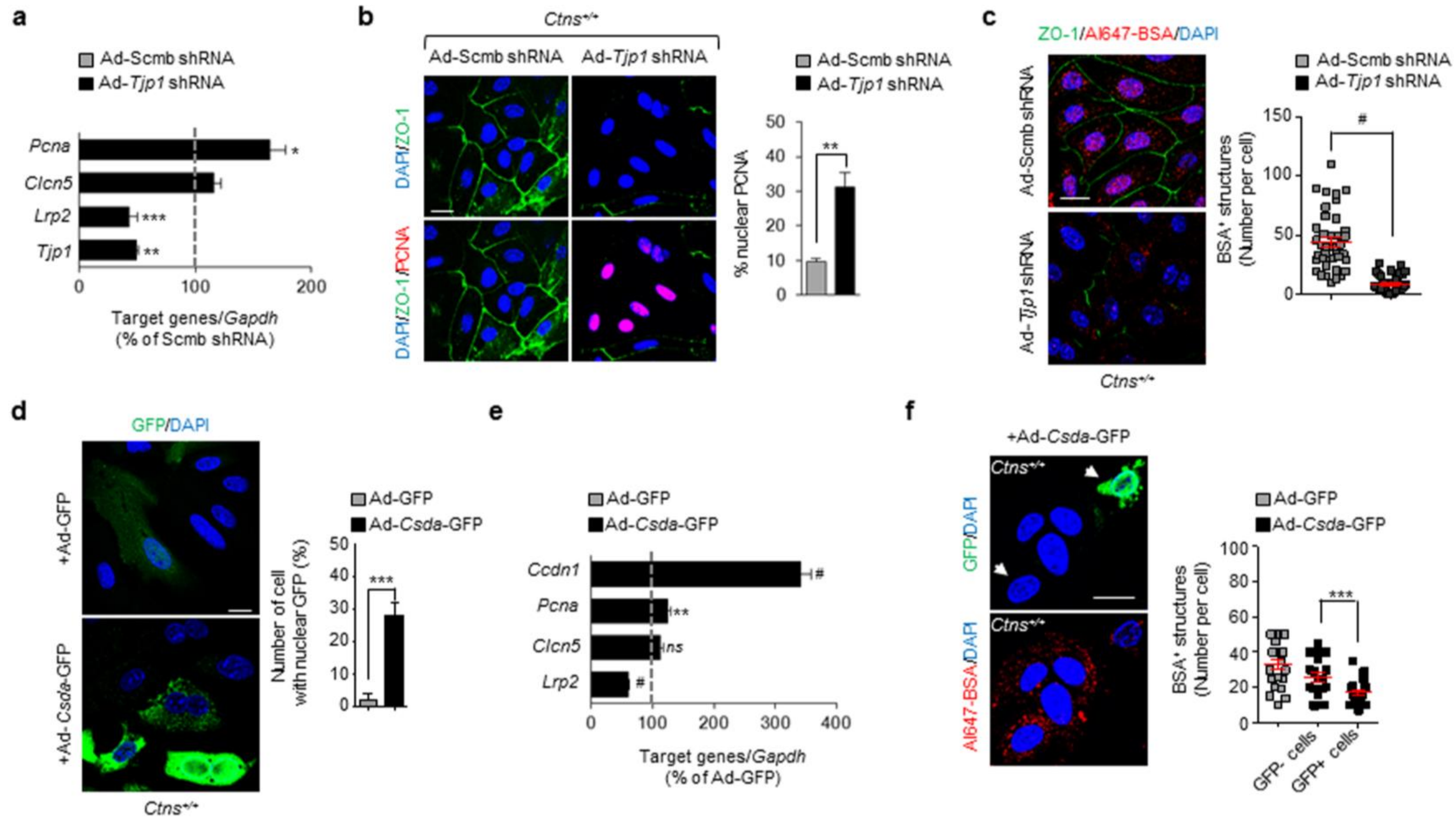

Supplementary Figure 10. Knockdown of *Tjp1* and the exogenous overexpression of *Csda* in PT cells promote epithelial dysfunction. (**a-c**) mPTCs were transduced with Scrambled (Scmb) or *Tjp1* adenoviral shRNAs for 5 days. (**a**) The mRNA levels of *Pcna, Clcn5, Lrp2 and Tjp1* were analysed by real-time PCR (n=4 independent experiments). (**b**) The cells were immunostained with anti-ZO-1(green) and anti-PCNA (red) antibodies and analysed by confocal microscopy. Quantification of the numbers PCNA$^+$ nuclei (expressed as percentage of the total nuclei) obtained from 5 randomly selected fields per condition, with each containing ~20-25 cells. (**c**) The cells were loaded with Al647-BSA (red; 50µg ml^{-1} for 15 min at 37°C), stained with anti-ZO-1 (green) antibody and analysed by confocal microscopy. Quantification of numbers of Al647-BSA$^+$ structures (n=50 randomly selected cells pooled from three mouse kidneys per condition; each point representing the number of BSA$^+$ structures in a cell). (**d-f**) mPTCs were transduced with Ad-GFP or GFP-tagged-(*Csda*)-bearing adenoviral particles for 2 days. (**d**) The cells were stained with anti-GFP antibody and analysed by confocal microscopy. Quantification of numbers of cells with nuclear GFP (expressed as percentage of the total nuclei; n=20 randomly selected cells per condition). (**e**) The mRNA levels of *Ccdn1, Pcna, Clcn5, Lrp2* were analysed by real-time PCR (n=4 independent experiments). (**f**) The cells were loaded with Al647-BSA (red; 50µg ml^{-1} for 15 min at 37°C), stained with anti-GFP antibody and analysed by confocal microscopy. Quantification of numbers of Al647-BSA$^+$ structures per cell (n=20 randomly selected cells pooled from three mouse kidneys per condition; each point representing the number of BSA$^+$ structures). Plotted data show mean ± SEM. Two tailed paired Student's *t* test, **P*<0.05, ***P*<0.01, ***P<0.001, and $^{\#}P$<0.0001 relative to *Ctns*$^{+/+}$ mPTCs transduced with Scmb shRNAs or with Ad-GFP vector or relative to GFP$^-$/*Ctns*$^{+/+}$ cells; *ns*, not significant. Nuclei are counterstained with DAPI (blue). Scale bars are 10µm.

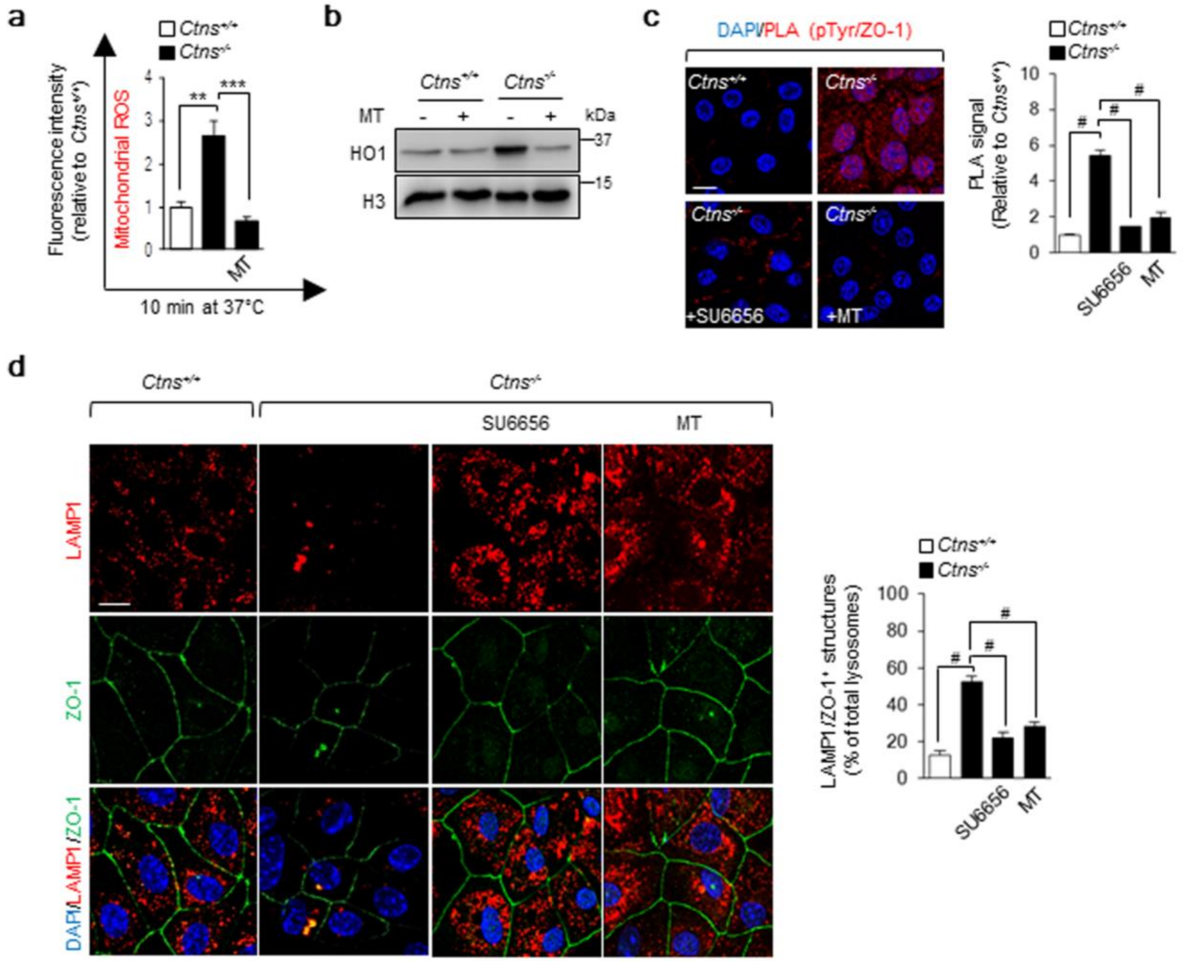

Supplementary Figure 11. Scavenging of mitochondrial ROS restores epithelial function in cystinosis PT cells. (**a-b**) *Ctns* mPTCs were cultured in presence or in absence of mitochondria-targeted antioxidant Mito-TEMPO (MT, 10µM for 24h). (**a**) The cells were loaded with MitoSOX (2.5µM for 10 min at 37°C) and analysed by confocal microscopy. Quantification of fluorescence intensity from 5 randomly selected fields per condition, with each containing ~20-25 cells; (**b**) Representative western blotting of HO1 protein levels. H3 was used as a loading control; n=2 independent experiments. (**c-d**) *Ctns* cells were treated with MT (10µM for 24h) or with a selective Src kinase inhibitor SU6656 (5µM for 24h). (**c**) Representative confocal micrographs and quantification of endogenous phosphorylation of ZO-1 by proximity ligation assay. (**d**) Cells were immunostained with anti-LAMP1 (red) and anti ZO-1 (green) antibodies, and analysed by confocal microscopy. Quantification of ZO-1/LAMP1^{+} structures (expressed as percentage of the total lysosomes) obtained from 3 randomly selected fields per condition, with each containing ~20-25 cells. Plotted data represent mean ± SEM. One–way ANOVA followed by Bonferroni *post hoc* test, $^{**}P<0.01$, $^{***}P<0.001$ and $^{\#}P<0.0001$ relative to untreated *Ctns*$^{+/+}$ or *Ctns*$^{-/-}$ mPTCs. Yellow indicates the colocalization in **d**. The nuclei are counterstained with DAPI (blue). Scale bars are 10µm.

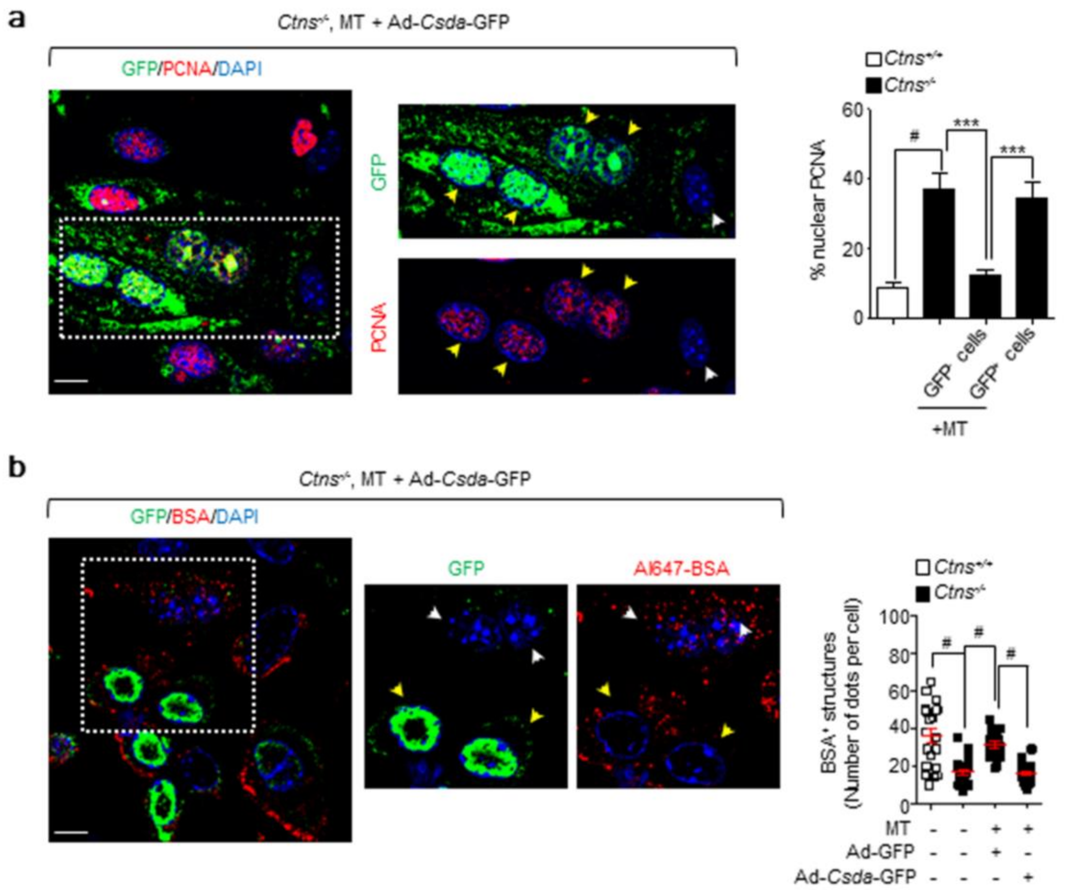

Supplementary Figure 12. *Csda* overexpression abolishes the effects of Mito-TEMPO on the epithelial function in cystinosis PT cells. (**a-b**) MT-treated *Ctns*$^{-/-}$ mPTCs were transduced with Ad-GFP or GFP-tagged-(*Csda*)-bearing adenoviral particles for 2 days. (**a**) The cells were immunostained with anti- PCNA (red) antibody and analysed by confocal microscopy. Quantification of PCNA^{+} nuclei (expressed as percentage of the total nuclei) obtained from 15 cells negative or positive for nuclear GFP enrichment. One-way ANOVA followed by Bonferroni *post hoc* test, $^{\#}P<0.0001$ relative to *Ctns*$^{+/+}$ or $^{***}P<0.001$ relative to *Ctns*$^{-/-}$ mPTCs or relative to GFP^{-}/*Ctns*$^{-/-}$mPTCs treated with MT. (**b**) The cells were loaded with Al647-BSA (red; 50µg ml^{-1} for 15 min at 37°C), stained with anti-GFP (green) antibody and analysed by confocal microscopy. Quantification of numbers of Al647-BSA^{+} structures per cell (n=20 cells negative or positive for nuclear GFP enrichment; each point representing the number of BSA^{+} structures in a cell). $^{\#}P<0.0001$ relative to *Ctns*$^{+/+}$ or *Ctns*$^{-/-}$ mPTCs or relative to Ad-GFP/*Ctns*$^{-/-}$ mPTCs treated with MT. Plotted data show mean ± SEM. Nuclei counterstained with DAPI (blue). Scale bars are 10µm.

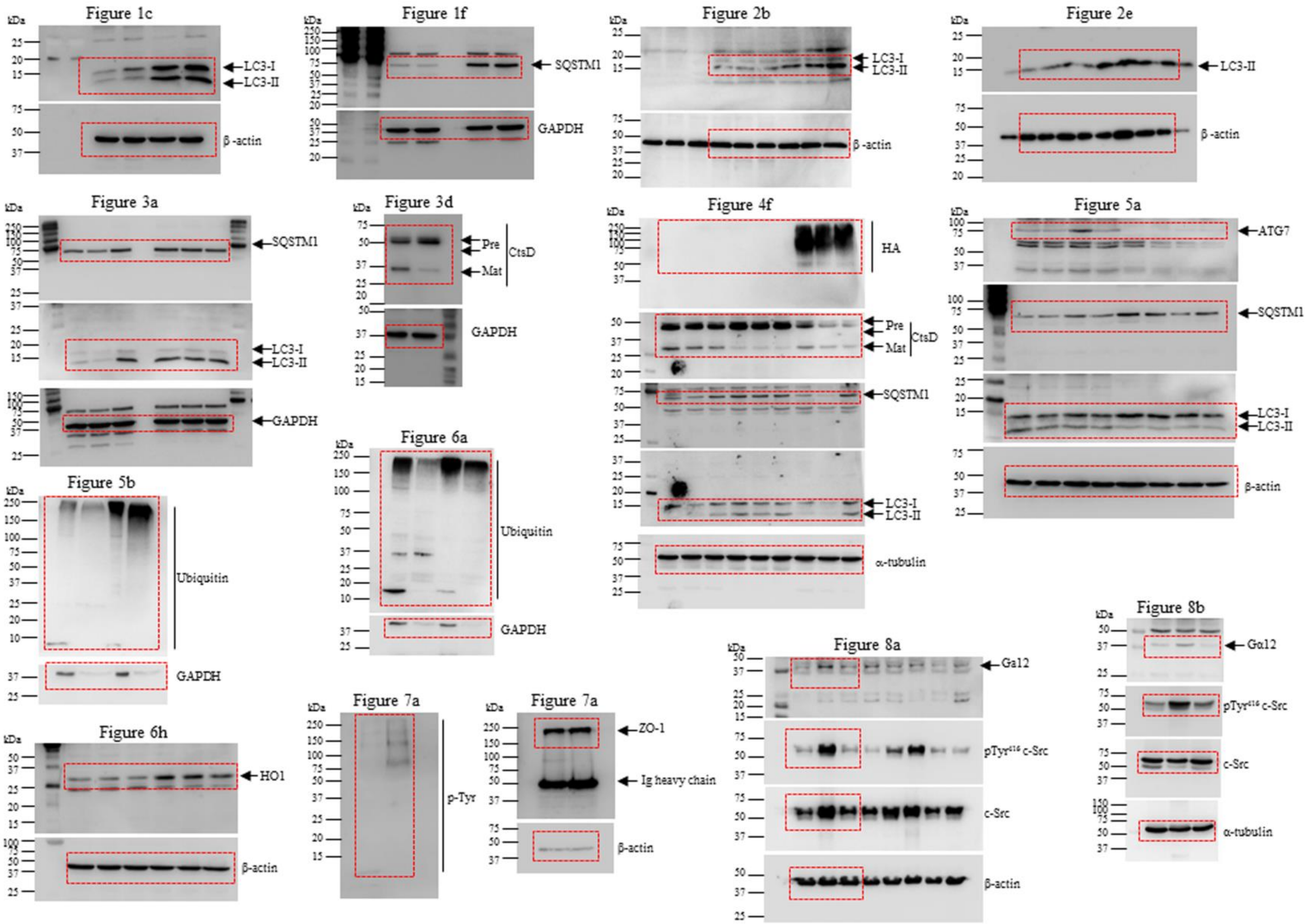

Supplementary Figure 13. Unprocessed and uncropped scans of original blots shown in figures and supplementary figures are reported.

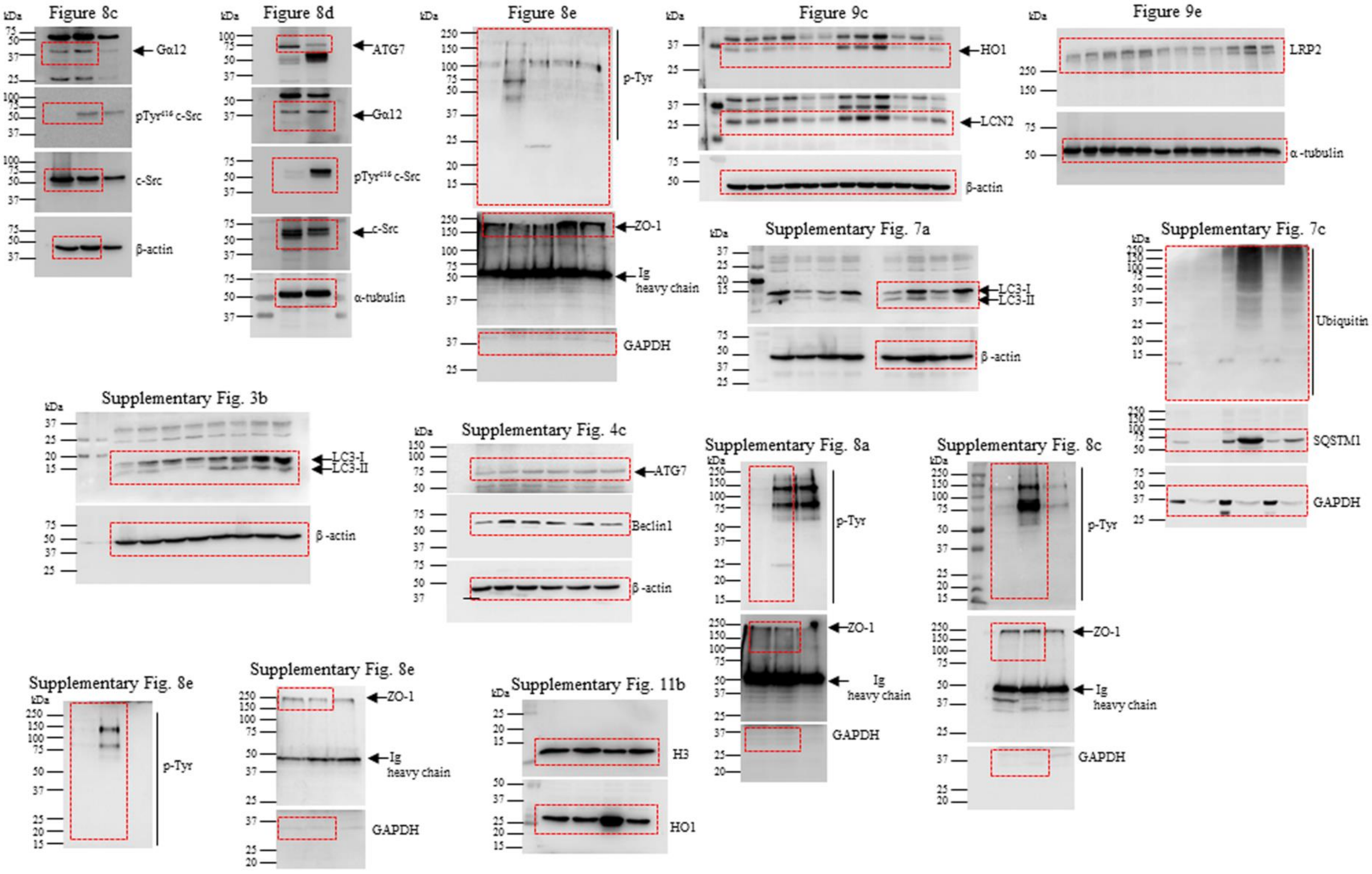

Supplementary Figure 13 (continued). Unprocessed and uncropped scans of original blots shown in figures and supplementary figures are reported.

Supplementary Table 1. List of the mouse primers used for gene expression analyses

Gene name	Forward primer (5'-3')	Reverse primer (5'-3')	PCR products (bps)	Efficiency
Gapdh	TGCACCACCAACTGCTTAGC	GGATGCAGGGATGATGTTCT	176	1.04 ± 0.03
Actb	TGCCCATCTATGAGGGCTAC	CCCGTTCAGTCAGGATCTTC	102	1.03 ± 0.04
Hprt1	ACATTGTGGCCCTCTGTGTG	TTATGTCCCCCGTTGACTGA	162	0.99 ± 0.01
Ppiase	CGTCTCCTTCGAGCTGTTTG	CCACCCTGGCACATGAATC	139	1.02 ± 0.02
18S	GTAACCCGTTGAACCCCATT	CCATCCAATCGGTAGTAGCG	151	0.98±0.02
36B4	CTTCATTGTGGGAGCAGACA	TTCTCCAGAGCTGGGTTGTT	150	1.02±0.02
Tjp1	CCACCTCTGTCCAGCTCTTC	TGGTGGTCTGAAAGTTGCTG	147	0.97±0.03
Lrp2	CAGTGGATTGGGTAGCAGGA	GCTTGGGGTCAACAACGATA	150	0.99±0.04
Pcna	TTGGAATCCCAGAACAGGAG	ATTGCCAAGCTCTCCACTTG	155	0.97±0.04
Ccnd1	AGCAGAAGTGCGAAGAGGAG	CAAGGGAATGGTCTCCTTCA	149	1.03±0.05
Atg7	AGCTTGGCTGCTACTTCTGC	CTGCAGGACAGAGACCATCA	149	0.99±0.03
Map1lc3a	CCTTCTTCCTGCTGGTCAAC	TGACTCAGAAGCCGAAGGTT	138	1.01±0.03
Map1lc3b	CCGAGAAGACCTTCAAGCAG	CCAGGAACTTGGTCTTGTCC	153	0.98±0.04
Becn1	AGGAGCTGGAAGATGTGGAA	ACTCCAGCTGCTGCCTTTTA	141	0.96±0.04
Slc34a1	CATCACAGAGCCCTTCACAA	TGGCCTCTACCCTGGACATA	161	1.02±0.03
Ybx-3/Csda	AGGACGCGGAGAAGAAAGTT	ACTTGCGTGGGTTGTTTTTC	153	0.98±0.04
Erbb2	TTTGTGGTCATCCAGAACGA	CAGGGTCTGGGGAGAAGAAT	151	0.97±0.03
Rab5	TGGGATACAGCTGGTCAAGA	AGGACTTGCTTGCCTTTGAA	153	0.98±0.03
Clcn5	TGGAGGAGCCAATCCCTGGTGT	AGAAAGCATCGCTCACACTG	156	1.01±0.03
Mki67	TGCAAAGGTAGAGGCTCCAT	CAGGTAGGCCAGAGCAAGT	152	1.00±0.02
Spp1	TCCAATCGTCCCTACAGTCG	CGCTCTTCATGTGAGAGGTG	146	1.02±0.04

Supplementary Table 2. List of the zebrafish primers used for gene expression analyses

Gene name	Forward primer (5’-3’)	Reverse primer (5’-3’)	PCR products (bps)	Efficiency
clcn7	GCAGAGTGTTCCTTTCCTCC	GACATCTACAATGGTTCCGAC	126	0.97 ± 0.04
ctns	TACCTTAGCAGCAATGACACTC	GCCACAAAGTAAATCCAGCCA	118	1.02 ± 0.03
actb1	TGAATCCCAAAGCCAACAGAG	TCACACCATCACCAGAGTCC	149	1.03 ± 0.04
