# Supplementary material for: Impaired autophagy bridges lysosomal storage disease and epithelial dysfunction in the kidney
Source: Nat Commun. 2018 Jan 11;9:161. doi: 10.1038/s41467-017-02536-7 (PMC5765140; doi:10.1038/s41467-017-02536-7)
Supplement: Supplementary file 2 — Description of Additional Supplementary Files [file 41467_2017_2536_MOESM2_ESM.pdf]

Description of Additional Supplementary Files

File Name: Supplementary Movie 1
Description: Confocal microscopy and three-dimensional (3D) reconstruction of $LAMP1^{+}$ vesicles in *Ctns*$^{+/+}$ mPTCs

File Name: Supplementary Movie 2
Description: Confocal microscopy and three-dimensional (3D) reconstruction of LAMP1+ vesicles in *Ctns*$^{-/-}$ mPTCs

File Name: Supplementary Movie 3
Description: Electron tomography micrographs and 3-D reconstructed tomograms showing the presence of enlarged, single membranous structures (reminiscent of autolysosome; green) filled with undegraded cellular debris (blue and pink) in *Ctns*$^{-/-}$ mPTCs
